# Hydrogen-bonded frameworks for molecular structure determination

**DOI:** 10.1038/s41467-019-12453-6

**Published:** 2019-10-02

**Authors:** Yuantao Li, Sishuang Tang, Anna Yusov, James Rose, André Nyberg Borrfors, Chunhua T. Hu, Michael D. Ward

**Affiliations:** Department of Chemistry and Molecular Design Institute, New York University, 100 Washington Square East, Room 1001, New York, NY 10003 USA

**Keywords:** X-ray diffraction, Structure elucidation, Crystal engineering

## Abstract

Single crystal X-ray diffraction is arguably the most definitive method for molecular structure determination, but the inability to grow suitable single crystals can frustrate conventional X-ray diffraction analysis. We report herein an approach to molecular structure determination that relies on a versatile toolkit of guanidinium organosulfonate hydrogen-bonded host frameworks that form crystalline inclusion compounds with target molecules in a single-step crystallization, complementing the crystalline sponge method that relies on diffusion of the target into the cages of a metal-organic framework. The peculiar properties of the host frameworks enable rapid stoichiometric inclusion of a wide range of target molecules with full occupancy, typically without disorder and accompanying solvent, affording well-refined structures. Moreover, anomalous scattering by the framework sulfur atoms enables reliable assignment of absolute configuration of stereogenic centers. An ever-expanding library of organosulfonates provides a toolkit of frameworks for capturing specific target molecules for their structure determination.

## Abstract (web summary)

Single crystal X-ray diffraction is an invaluable tool for molecular structure determination, but growing single crystals is often an arduous process. Here the authors find that the structures of a wide array of molecules can be determined by SCXRD when included in hydrogen-bonded guanidinium organosulfonate host frameworks in a single-step crystallization.

## Introduction

The centrality of molecular structure to chemistry cannot be disputed, dating back to the pioneering efforts of 19th century scientists such as Jacobus Henricus van’t Hoff^1^, Louis Pasteur^2,3^ and Hermann Emil Fischer^4^, who collectively formulated the early principles of stereochemistry that guide contemporary organic chemistry. Although an arsenal of methods now exists for structure determination, including NMR spectroscopy^5–8^, X-ray crystallography remains the most definitive, particularly for molecules with one or more stereogenic centers requiring assignment of absolute configuration. Johannes Martin Bijvoet first reported the determination of absolute configuration of sodium rubidium (+)-tartrate tetrahydrate using single crystal X-ray diffraction^9^, wherein anomalous X-ray scattering by the rubidium heavy atoms enabled definitive structure determination^10^. Molecular structure determination by single-crystal X-ray diffraction, however, often can be frustrated by the inability to grow sufficiently large single crystals for conventional X-ray diffraction analysis, the tendency of many compounds to form oils or amorphous phases, low melting points that preclude solidification at convenient temperatures, and decomposition under ambient conditions. In the case of chiral molecules, insufficient anomalous scattering due to the absence of heavy elements also can preclude accurate absolute configuration determination.

Molecular structure determination of such stubborn target molecules has been realized by single-crystal X-ray diffraction following their adsorption into a limited number of metal organic frameworks^11–15^ and others (a.k.a. crystalline sponges)^16,17^. Co-crystallization explicitly for determination of the molecular structure of compounds that otherwise cannot be crystallized relies on specific interactions, and it is limited by an absence of universal co-crystallizing agents^18,19^. The crystalline sponge approach has been used for absolute configuration determination for natural products, synthetic molecules, and reaction intermediates^20–24^. Target molecules also have been trapped reactively by a chiral metal-organic framework, wherein framework formate ligands are exchanged with carboxylate or hydroxyl groups of the targets^25^. The crystalline sponge method (CSM) is undeniably innovative, and under certain conditions it requires only minute amounts of the target compound (<1 µg). CSM, however, often requires specific intermolecular host-guest interactions or covalent fixation and is hindered by slow absorption kinetics that can require weeks for complete target incorporation, the need to use non-polar solvents, an upper size limit on target molecules imposed by the size of the pore apertures, and challenges in structure determination presented by disorder, partial occupancy and retention of solvent molecules in the framework cages.

**Fig. 1:**
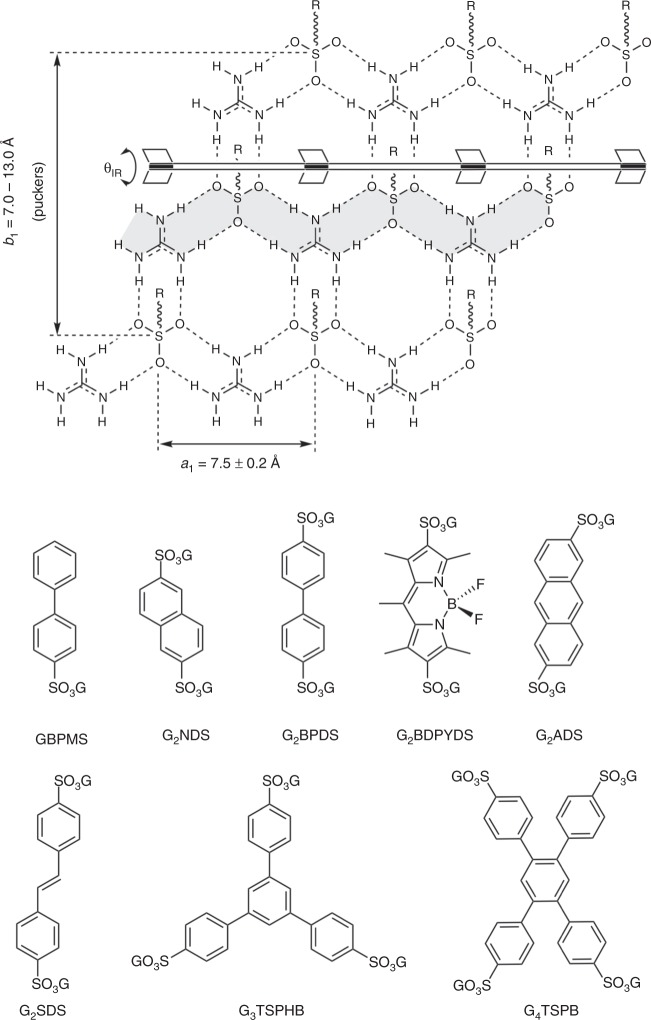
GS framework components. Top: the typical quasi-hexagonal GS sheet, illustrating the hydrogen-bonded hinge between hydrogen-bonded major ribbons (one of these is highlighted in gray) that allows facile puckering of the GS sheet with concomitant shrink-wrapping around guests in the inclusion cavities. The R substituents on the sulfur atoms denote organic groups that can project from either side of the GS sheet. Bottom: Guanidinium organosulfonates used here

Our laboratory has reported a substantial number of molecular frameworks, built from two-dimensional hydrogen-bonded networks of guanidinium (G) and organosulfonate (S) ions (Fig. 1), capable of encapsulating a wide range of guests^26–30^. The large number of synthetically accessible organosulfonates, combined with the innate ability of the frameworks to adopt various architectures, enables inclusion of a wide range of guest sizes and shapes without a requirement for specific interactions. Moreover, the frameworks are inherently compliant because of the flexibility of the N–H…O–S hydrogen bonds, which allows the framework to shrink wrap around guest molecules by puckering of the GS network about a hinge with retention of hydrogen bond connectivity. This attribute eliminates disorder and mitigates solvent inclusion in the vast majority of cases. More than five hundred inclusion compounds with well-characterized single crystal structures, including the structures of the confined guests, have been realized. Moreover, GS frameworks contain sulfur atoms, which can provide strong anomalous scattering for determination of relative stereochemistry and absolute configuration of stereogenic centers in guest molecules consisting of light atoms^31–33^. Herein we demonstrate a convenient approach that complements the CSM in which target molecules are encapsulated by the highly versatile hydrogen-bonded frameworks of guanidinium (G) and organosulfonate (S) ions in a single-step crystallization, in the absence of specific interactions and in stoichiometric amounts, enabling facile determination of molecular structure and absolute configuration.

## Results

### Structure determination using GS frameworks

**Fig. 2:**
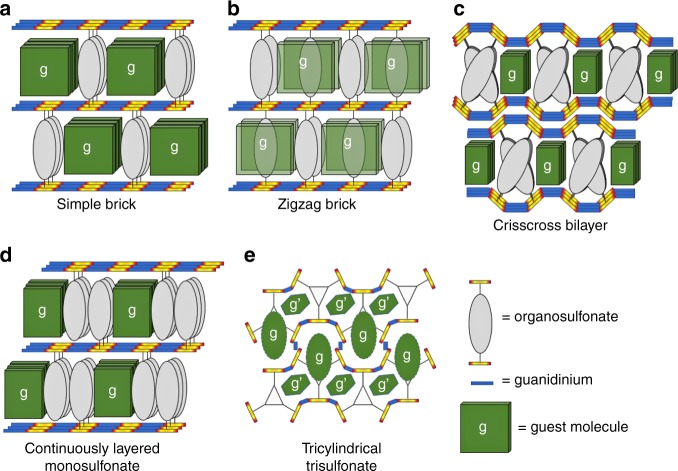
Schematic representations of host frameworks appearing herein. **a**–**c** Guanidinium disulfonate simply brick, zigzag brick, and crisscross bilayer architectures. **d** Guanidinium monosulfonate continuously layered architecture. **e** Tricylindrical architecture observed for the guanidinium tri(4-sulfophenyl)benzene host (G_3_TSPHB)

A GS host for a particular guest can be selected by choosing organosulfonates capable of forming inclusion cavities sufficiently large to accommodate the guest molecule. Recognizing that custom fitting is not a precise science, guest inclusion is best achieved using a collection of organosulfonates that bracket the required inclusion volume in the various possible framework architectures (Fig. 2, Supplementary Fig. 1, Supplementary Table 1). Guanidinium monosulfonates also form inclusion compounds readily despite the absence of enforced cavities, and they are not constrained by the requirement for registry between opposing GS sheets^34^.

**Fig. 3:**
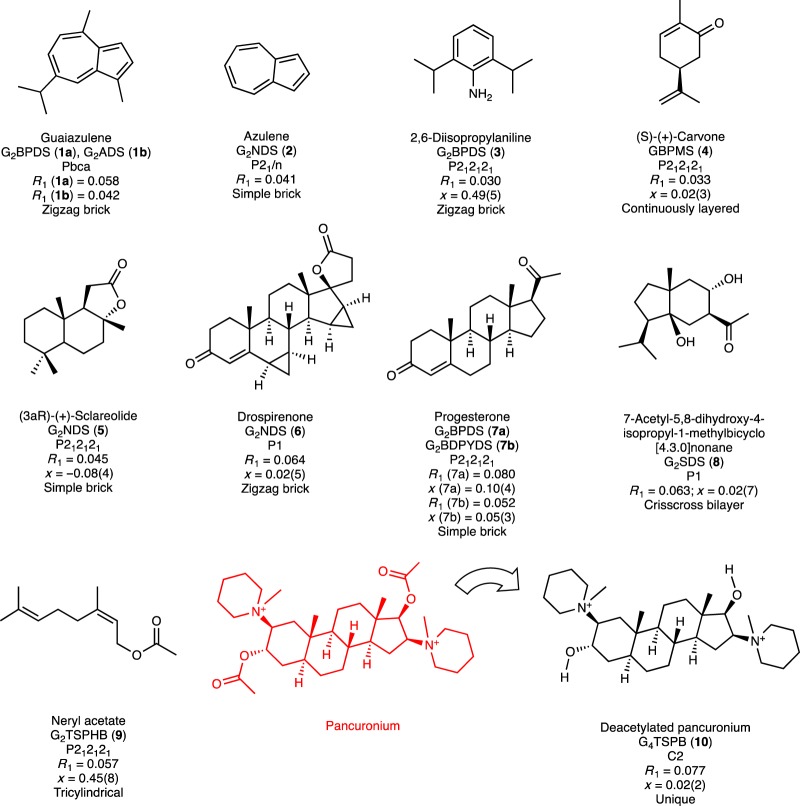
Target molecules, in GS frameworks, characterized by single crystal X-ray diffraction. The GS framework(s) corresponding to each target molecule and the *R*_1_ values for observable reflections are provided. Flack parameters (*x*) are given for compounds that crystallized in chiral space groups. Values of *x* near 0.5 observed for achiral guests are consistent with twinning. The near-zero value and high precision of the Flack parameters (*x*) for inclusion compounds with chiral guests provides confidence in the assignment of absolute configuration^44^. The deacetylated pancuronium was trapped as a hydrolysis product of pancuronium during crystallization, but without a conventional GS framework

Target molecules can be included within the GS host frameworks in stoichiometric quantities through a single-step crystallization in which the target molecule is simply added to a solution of the GS framework components. Guaiazulene, a pigment with a melting point close to room temperature found in soft corals and mushrooms, was used as a benchmark for comparison with the crystalline sponge method^12–14^ (Fig. 3, Supplementary Tables 3 and 4). MM2 calculations performed to calculate the guaiazulene volume suggested the simple brick and zigzag architectures of G_2_BPDS, G_2_ADS, and G_2_SDS would be suitable for guaiazulene inclusion. Indeed, inclusion compounds of (G_2_BPDS) ⊃ (guaiazulene)_2_ (**1a**) and (G_2_ADS) ⊃ (guaiazulene)_2_ (**1b**) (the ⊃ symbol denotes inclusion) formed well-defined single crystals (Supplementary Fig. 2) in a single step within 24 h by slow evaporation of methanol solutions containing guaiazulene and either of the two hosts. Single-crystal X-ray diffraction data determined that compound **1a** crystallized in the orthorhombic *Pbca* space group (Supplementary Table 2), revealing the zigzag brick architecture and pairs of guaiazulene guests trapped in pockets flanked by the organic pillars (Supplementary Fig. 16). The guaiazulene guests were disordered, but the four disordered components could be modeled and refined unambiguously. Compound **1b** crystallized in the same space group and architecture (Supplementary Fig. 17), but the guaiazulene guests were only slightly disordered, with two guaiazulene components in a 93:7 ratio. The molecular structure of the major disordered component was refined freely without any restraints and constraints. This illustrates that disorder can be reduced substantially with a suitable host, in this case through the use of a conformationally rigid organosulfonate pillar. Azulene, which is considerably smaller than guaiazulene, was included in the smaller channel-like cavities of the G_2_NDS simple brick framework, as (G_2_NDS) ⊃ (azulene)_3_ (**2**) (Supplementary Fig. 18). The compound 2,6-diisopropylaniline, another CSM benchmark (Supplementary Tables 3 and 4), was included in the pockets of the G_2_BPDS zigzag brick framework, to afford block shaped single crystals of (G_2_BPDS) ⊃ (2,6-diisopropylaniline)_2_ (**3**) and refined in the *P*2_1_2_1_2_1_ space group (Supplementary Fig. 19).

(S)-(+)-carvone is a relatively simple natural compound containing one chiral center, but its low melting point (25.2 °C) frustrates determination of its molecular structure by single crystal X-ray diffraction in the absence of inclusion by a host^35,36^. This compound was included in G_2_BPDS and G_2_ADS hosts by single-step crystallization to afford clusters of wispy crystalline needles, but the guanidinium monosulfonate GBPMS formed well-defined single crystals of (G_2_BPMS) ⊃ ((S)-( + )-carvone) (**4**) that were more amenable to single crystal structure analysis. Compound **4** crystallized in the space group *P*2_1_2_1_2_1_ with the continuously layered architecture (Supplementary Fig. 20), which resembles the simple brick architecture of guanidinium disulfonates. The Flack parameter – a measure of the reliability of the assignment of absolute configuration – is provided in Fig. 3 beneath compound **4** and all other chiral entries. In each case the Flack parameter is well within an acceptable threshold for reliable assignment of absolute configuration, reflecting the contribution of anomalous scattering expected from the sulfur atoms.

**Fig. 4:**
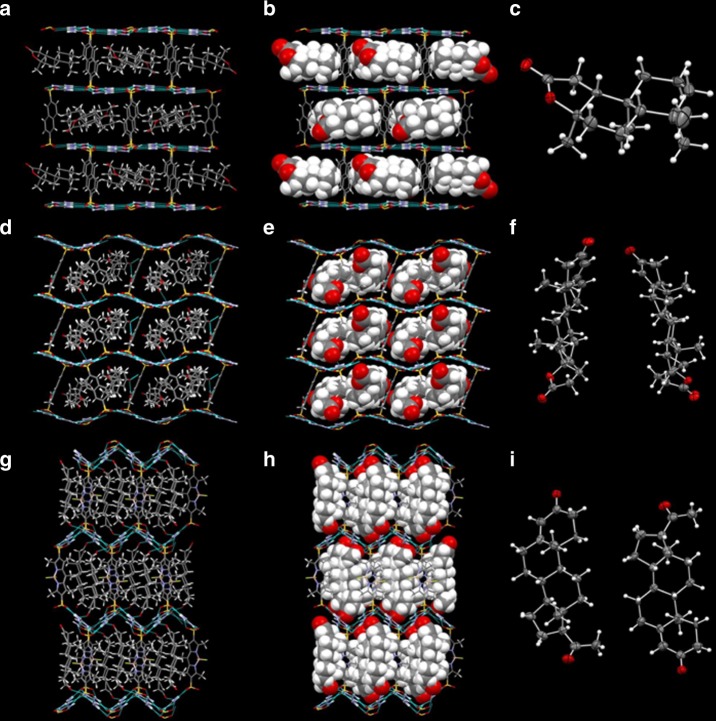
Illustrative crystal structures of GS inclusion compounds. These are depicted as stick figures that illustrate the 100% occupancy (left panel), the target guest molecules as space filling (center), and ORTEP representations of the guests. **a**–**c** (G_2_NDS) ⊃ (3aR)-( + )-Sclareolide (**5**); **d**–**f** (G_2_NDS) ⊃ (drospirenone)(methanol)_0.84_(H_2_O)_0.1_ (**6**); **g**–**i** (G_2_BDPYDS) ⊃ (progesterone) (**7b**). The stick renderings in the leftmost panels reveal the 100% occupancy of target guest molecules in the inclusion cavities and the center panel illustrates the substantial contribution of the guests to the total crystal volume

The single-step crystallization approach is further illustrated by the crystallization of GS inclusion compounds with guests having multiple stereogenic centers (Fig. 4). (3aR)-(+)-Sclareolide, bearing four chiral centers, is a sesquiterpene lactone natural product used as a fragrance in the cosmetics industry. Single-step crystallization from methanol produced single crystals of (G_2_NDS) ⊃ ((3aR)-(+)-Sclareolide) (**5**) in the space group *P*2_1_2_1_2_1_ (Supplementary Fig. 21), with the (3aR)-(+)-Sclareolide guests arranged with their long axes aligned with the channels of the simple brick framework. The rectangular channels of the G_2_NDS pillars conformed to the shape of guest molecules, and no disorder was observed. Drospirenone, a progestin used in birth control drugs, is significantly larger than (3aR)-(+)-Sclareolide. Nevertheless, crystallization with G_2_NDS from methanol produced single crystals of (G_2_NDS) ⊃ (drospirenone)(methanol)_0.84_(H_2_O)_0.1_ (**6**). The structure refined in the space group *P*1 and adopted the zigzag brick architecture, which has larger cavities than the brick framework^37^ (Fig. 4, Supplementary Fig. 22). The Flack parameters for compounds **5** and **6** were very precise, allowing assignment of the absolute configuration of the four stereogenic centers in each compound with high confidence. Notably, the absolute configurations of (3aR)-(+)-Sclareolide and drospirenone have not been established by X-ray diffraction (Supplementary Table 5)^38,39^.

Progesterone, another progestin commonly known as the female sex hormone, crystallized with G_2_BPDS to produce (G_2_BPDS) ⊃ (progesterone)(ethanol) (**7a**), forming needle-shaped crystals that were refined in the orthorhombic space group *P*2_1_2_1_2_1_. The G_2_BPDS framework adopted the simple brick architecture with a highly puckered GS sheet. The long axes of the progesterone guests were aligned with channels flanked by the BPDS pillars, with pairs of progesterone molecules spanning 26.3 Å, commensurate with the sulfonate nodes along the major GS ribbon (Supplementary Fig. 23). This example, along with compound **6**, illustrates that satisfactory refinements can be obtained even if solvent is included along with the target molecule. Unlike solvent that persists in crystalline sponges, included solvent in GS compounds is typically stoichiometric and readily refined. Solvent inclusion can be eliminated completely, however, by using the slightly larger BDPYDS pillar, which crystallized as G_2_BDPYDS ⊃ (progesterone) (**7b**), in the same space group and framework architecture as **7a**, but with improved refinement (Supplementary Fig. 24). The long axes of the progesterone guests in **7b** were perpendicular to the channels along the major GS ribbon axis. The volume of BDPYDS is 80 Å^3^ greater than that of BPDS, an amount similar to the volume of the ethanol molecule in **7a** (50 Å^3^), which explains the absence of solvent inclusion. Despite the loss of one N–H…S–O hydrogen bond owing to a competing N–H…O = C(progesterone) hydrogen bond, the quasi-hexagonal motif of the GS sheet remains intact. The adaptability of the GS frameworks to various guests is illustrated further by compound (G_2_SDS) ⊃ (7-Acetyl-5,8-dihydroxy-4-isopropyl-1-methylbicyclo[4.3.0]nonane)_0.5_ (**8**), in which the guest molecules are confined within channels of a crisscross bilayer architecture wherein the sulfonate nodes of 4,4′-stilbenedisulfonate pillars alternate on adjacent major ribbons along each channel (Supplementary Fig. 25).

The G_3_TSPHB and G_4_TSPB frameworks are less conventional and more constrained with respect to architectural diversity owing to their polyvalency. Nonetheless, G_3_TSPHB included the conformationally flexible neryl acetate, as well as isophorone guests, to form (G_3_TSPHB) ⊃ (neryl acetate)(isophorone)_3_ (**9**). In this example, isophorone assists the formation of the inclusion compound, similar to previous observations^40,41^. The structure of **9** was refined in the orthorhombic space group *P*2_1_2_1_2_1_ (Supplementary Fig. 26) with the guests confined within three crystallographically unique one-dimensional channels and between the central benzene ring. One of the channels is characterized by walls comprising four major GS ribbons and the concave surfaces of the TSPHB pillars whereas the other channel comprises two opposing GS ribbons and two opposing concave surfaces of the pillars. Despite the complexity of the framework structure, the GS sheet maintains its quasi-hexagonal motif but with a serpentine contour.

Interestingly, the addition of pancuronium bromide, a dicationic aminosteroid muscle relaxant, to a methanol solution containing guanidinium tetra(4-sulfophenyl)benzene (G_4_TSPB, followed by slow evaporation at 40 °C, afforded single crystals of G_6_(TSPB)_2_ ⊃ (deacetylated pancuronium) (H_2_O)_3.08_ (**10**). The crystal structure was refined in the monoclinic space group *C*2 (Supplementary Fig. 27), but without the typical GS network or well-defined channels (Supplementary Figs. 28 and 29) observed previously for this host^30^. Instead, the host forms a complex framework held together by N–H…O–S hydrogen bonds combined with water bridging G and S ions by hydrogen bonding, resulting in pockets occupied by the guests. Notably, the two acetyl groups of pancuronium were hydrolyzed but with retention of stereochemistry, demonstrating that crystallization can capture reaction products in situ.

## Discussion

These results demonstrate that the GS frameworks are inherently versatile with respect to molecular structure characterization by single-crystal X-ray diffraction following a simple single-step crystallization. The aforementioned examples demonstrate several important features of this approach, including (i) 100% occupancy of the target guest molecules, which leads to roughly half the volume occupied by the guest and enables straightforward, high quality structure refinement; (ii) access to different framework architectures and the availability of innumerable organosulfonates, which can accommodate the steric needs of the target guests; (iii) the ability to include a wide range of guests, from non-polar to polar, from aliphatic to aromatic, with many guest functional groups tolerated; (iv) shrink-wrapping of the hosts around the guests, through GS sheet puckering and conformational freedom of the pillars, which minimizes, or eliminates, the occurrence of disorder and solvent incorporation; (v) reduction or elimination of disorder through alternative organosulfonate pillars; (vi) anomalous scattering supplied by the sulfur atoms that provides for reliable assignment of absolute configuration, whether a copper or molybdenum source is used. Moreover, the amount of target material required for a single crystal amenable to conventional structure analysis can be as little as several micrograms. Crystallization of **5**, for example, was achieved by adding only 5 μg of (3aR)-(+)-sclareolide to 1 μL methanol containing 8 μg G_2_NDS in the tip of a 1-mL conical vial. The actual amount of guest in each crystal was ~0.1 µg, suggesting even lesser amounts of target guest could be used with smaller crystallization volume^42,43^. Collectively, these attributes provide for a convenient toolkit for the synthetic chemistry community.

## Methods

### Crystallization protocol

Organosulfonates for a particular guest were selected based on calculation of the molecular volumes of the target guest molecules using BIOVIA Materials Studio 2018 using Connolly surfaces in the Volume function (Supplementary Table 1). Single crystals of the inclusion compounds containing target molecules were typically obtained by dissolving a target molecule and a guanidinium organosulfonate (GS) apohost in a suitable solvent in a small vial, followed by slow evaporation at ambient temperature. Single crystals of inclusion compounds typically formed within 36 h, although some required as much as 10 days. Details are provided for each compound in the Supplementary information. The single crystals were retrieved from the crystallization medium and mounted on a 0.2-mm MicroMount (MiTeGen) with Type B immersion oil (Cargille Labs). Single-crystal X-ray diffraction data were obtained using a Bruker SMART APEX II diffractometer equipped with a CCD detector. The X-ray beam generated from a sealed Mo tube (*λ* = 0.71073 Å) was monochromated by a graphite crystal and collimated by a MonoCap collimator. Data were collected at (100 K) with an Oxford Cryosystems 700 + Cooler and processed using the APEX2 software for data reduction, data correction and cell refinement. Crystal structures were solved by SHELXT and refined with full-matrix least squares by SHELXL. Non-hydrogen atoms were refined with anisotropic displacement parameters, and hydrogen atoms were placed in idealized positions and refined with riding models.

## Supplementary information

Supplementary Information

## Data Availability

The X-ray crystallographic coordinates for structures reported in this study have been deposited at the Cambridge Crystallographic Data Centre (CCDC). CCDC numbers for compounds **1a** – **10** are 1867926–1867936, 1905338 (**7b**), and 1867937 (**5micro**). These data can be obtained free of charge from The Cambridge Crystallographic Data Centre via www.ccdc.cam.ac.uk/data_request/cif.
