# Supplementary material for: Hydrogen-bonded frameworks for molecular structure determination
Source: Nat Commun. 2019 Oct 2;10:4477. doi: 10.1038/s41467-019-12453-6 (PMC6775153; doi:10.1038/s41467-019-12453-6)
Supplement: Supplementary file 1 — Supplementary Information [file 41467_2019_12453_MOESM1_ESM.pdf]

A hydrogen-bonded framework toolkit for molecular structure determination

Yuantao Li, Sishuang Tang, Anna Yusov, James Rose, André Nyberg Borrfors, Chunhua T. Hu*, Michael D. Ward*

Department of Chemistry and the Molecular Design Institute, New York University, 100 Washington Square East, New York, NY 10003. e-mail: ch98@nyu.edu; mdw3@nyu.edu

Supplementary information

Contents

Supplementary Methods

Supplementary Tables

Supplementary Table 1. Structural features of inclusion compounds **1a** - **9**.

Supplementary Table 2. Crystallographic data for compounds **1a** – **10**.

Supplementary Table 3. Comparison of the structure refinement for guaiazulene and 2,6-diisopropylaniline using the single-step crystallization GS framework method, crystalline sponge method (CSM) and the improved crystalline sponge method (CSM improved).

Supplementary Table 4. Comparison of bond lengths of compounds **1a**, **1b**, **3** with their crystalline sponge counterparts.

Supplementary Table 5. Previously reported single crystal data of guest molecules discussed in the article.

Supplementary Figures

Supplementary Figure 1. Information of target molecules included in GS frameworks.

Supplementary Figure 2. Images of single crystals of compounds **1a** - **10**.

Supplementary Figure 3 – 15. Molecular structure of **1a** – **10** depicted as ellipsoids.

Supplementary Figure 16 - 27. Crystal structures of **1a** – **10**.

Supplementary Figure 28. GS hydrogen-bonded sheet motifs of compounds **1a** – **9**.

Supplementary Figure 29. Hydrogen bonds of guanidinium sulfonate and water in compound **10**.

Supplementary Figure 30. Image of single crystal (G_2NDS)⊃((3aR)-(+)-Sclareolide) grown from 5 micrograms of (3aR)-(+)-Sclareolide.

Supplementary References

Supplementary Methods

Materials. Isophorone was purchased from Tokyo Chemical Industry Co., Ltd (Tokyo, Japan). Na_2BDPYDS was purchased from Exciton (Mason, OH). Other starting materials were purchased from Sigma-Aldrich (St. Louis, MO). The various organosulfonic acids or their metal salt were purchased or synthesized using standard procedures.[1,2,3,4,5] Metal salts of the sulfonic acids were converted to the acid form by passing them through an Amberlyst 36 ion-exchange column. Guanidinium organosulfonate apohosts were prepared by combining acetone solutions of approximately 1.10 molar equivalents of guanidinium tetrafluoroborate and 1.0 molar equivalents of a select organosulfonic acid to produce a precipitate of the corresponding guest-free apohost of the GS compound.[6] Alternatively, 1.10 equivalents guanidinium tetrafluoroborate was added to an aqueous solution containing 1.0 equivalents of a select organosulfonic acid. The mixture was dried in a rotary evaporator and the resulting solid washed with acetone several times and dried *in vacuo*, affording the apohost of the GS compound.

Single crystal X-ray diffraction. Single crystal X-ray diffraction data was obtained using a Bruker SMART APEX II diffractometer equipped with a CCD detector. The X-ray beam generated from a sealed Mo tube was monochromated by a graphite crystal and collimated by a MonoCap collimator. The wavelength from the Mo Kα radiation is 0.71073 Å. Crystal temperature (100 K) was controlled by an Oxford Cryosystems 700+ Cooler. Crystals were mounted on a 0.2 mm MicroMount (MiTeGen) with Type B immersion oil (Cargille Labs). The structures were solved by SHELXT[7] and refined with full-matrix least squares by SHELXL (Sheldrick 2014).[8] Non-hydrogen atoms were refined with anisotropic displacement parameters, and hydrogen atoms were placed in idealized positions and refined with riding models. Crystallographic data of these structures, including cif, fcf, and hkl files, have been deposited with the Cambridge Crystallographic Data Centre. Copies of these data can be requested, free of charge, from the CCDC website at https://www.ccdc.cam.ac.uk/structures/.

Crystallization methods. Single crystals of inclusion compounds **1**-**10** were obtained by evaporation of the solvent containing the target guest molecule and the guanidinium organosulfonate (GS) apohost. Crystallization was typically performed in a 20 mL scintillation vial, which is useful when more than 1 mg of target molecule is available. A 5 mL borosilicate glass test tube was used when less than 1 mg of target guest molecule was available. Single crystals of the inclusion compounds were typically achieved by slow evaporation of the solvent under ambient temperature over a period of one to five days. **Crystallization of G_2BPDS⊃(guaiazulene)$_2$ (1a).** Slow evaporation under ambient conditions of 0.5 mL methanol solution containing 5 mg (25 μmol) of guaiazulene and 10 mg (23 μmol) of (guanidinium)$_2$ (4,4'-biphenyldisulfonate) (G_2BPDS) apohost in a 20 mL scintillation vial afforded blue block-shaped single crystals within 24 hours with the formula (G_2BPDS)⊃(guaiazulene)$_2$ (**1a**). NMR (400 MHz, d_6-DMSO): 8.08 (s, 2H), 7.59 – 6.94 (m, 16H), 6.84 (s, 12H), 3.10 (m, 2H), 2.76 (s, 6H), 2.51 (s, 6H), 1.13 (d, 12H). Single crystal X-ray diffraction determined that the compound crystallized in the *P*bca space group with the zigzag brick architecture. The guaiazulene target was slightly disordered, with four components in a ratio of 44%:25%:21%:10%. The molecular structure of the major disordered component was refined freely without any restraints and constraints.

Crystallization of G_2ADS⊃(guaiazulene)$_2$ (1b). Slow evaporation under ambient conditions of 0.5 mL methanol solution containing 5 mg (25 μmol) of guaiazulene and 10 mg (22 μmol) of (guanidinium)$_2$(2,6-anthraceneldisulfonate) (G_2ADS) apohost afforded blue plate-shaped crystals within 24 hours with the formula (G_2ADS)⊃(guaiazulene)$_2$ (**1b**). NMR (400 MHz, d_6-DMSO): 8.60 (s, 2H), 8.17 (s, 2H), 8.09 (s,

2H), 7.93 (s, 2H), 7.00-7.59 (m, 10H), 6.81 (s, 12H), 3.10 (m, 2H), 2.76 (s, 6H), 2.60 (s, 6H), 1.13 (m, 12H). Single crystal X-ray diffraction determined that the compound crystallized in the *P*bca space group with the zigzag brick architecture. The guaiazulene target was slightly disordered, with two components in a 93:7 ratio. The molecular structure of the major disordered component was refined freely without any restraints and constraints.

Crystallization of $G_2NDS \supset (azulene)_3$ (2). Slow evaporation under ambient conditions of 1 mL of methanol containing 5 mg (39 μmol) of azulene and 10 mg (25 μmol) of (guanidinium)$_2$(2,6-naphthaleneldisulfonate) (G_2NDS) apohost afforded thin blue plates within 36 hours having the formula (G_2NDS)⊃(azulene)$_3$ (**2**). NMR (400 MHz, *d*$_6$-DMSO): 8.44 (d, 6H), 8.11 (s, 2H), 7.91 (t, 3H), 7.90 (d, 2H), 7.70 (d, 2H), 7.69 (t, 3H), 7.41 (d, 6H), 7.24 (t, 6H), 6.90 (s, 12H). Single crystal X-ray diffraction determined that the compound crystallized in the $P2_1/n$ space group with the simple brick architecture. Despite slight disorder, the molecular structure of the azulene target was refined satisfactorily and the structure found identical to its previously reported structure.

Crystallization of $G_2BPDS \supset$(2,6-diisopropylaniline)$_2$ (3). Slow evaporation under ambient conditions of 2 mL of methanol containing 80 μL (75 mg, 0.42 mmol) of 2,6-diisopropylaniline and 5 mg (12 μmol) of (guanidinium)$_2$(4,4'-biphenyldisulfonate) (G_2BPDS) apohost afforded colorless block-shaped single crystals within ten days having the formula (G_2BPDS)⊃(2,6-diisopropylaniline)$_2$ (**3**). NMR (400 MHz, *d*$_6$-DMSO): 7.52 (q, 8H), 6.82 (s, 12H), 6.76 (d, 4H), 6.46 (t, 2H), 4.40 (s, 4H), 2.90 (m, 4H), 1.03 (d, 24H). Single crystal X-ray diffraction determined that the compound crystallized in the $P2_12_12_1$ space group with the zigzag brick architecture. The structure was refined successfully with no constraints, consistent with dense packing of guest molecules in the framework.

Crystallization of GBPMS⊃(S-(+)-carvone) (4). Slow evaporation for two days under ambient conditions of 1 mL of methanol containing 50 μL (48 mg, 0.32 mmol) of S-(+)-carvone, a terpenoid found in caraway seeds, and 5 mg (17 μmol) of (guanidinium)$_2$(4-biphenylsulfonate) (GBPMS) apohost afforded colorless needle-shaped single crystals having the formula (GBPMS)⊃(S-(+)-Carvone) (**4**). NMR (400 MHz, *d*$_6$-DMSO) 7.70-7.61 (m, 4H), 7.49-7.35 (m, 5H), 6.92 (s, 6H), 6.85 (m, 1H), 4.76 (d, 2H), 2.64-2.28 (m, 5H), 1.70 (m, 3H), 1.67 (m, 3H). Single crystal X-ray diffraction determined that the compound crystallized in the $P2_12_12_1$ space group with the S-(+)-Carvone guest aligned along the inclusion channels in the simple continuous layered architecture. The Flack parameter, which reflects the correctness of chirality assignment, was found to be 0.02(3), indicating definitive determination of absolute configuration.

Crystallization of $G_2NDS \supset$((3aR)-(+)-sclareolide) (5). Slow evaporation under ambient conditions of 2 mL of methanol containing 6 mg (24 μmol) of (3aR)-(+)-sclareolide, a sesquiterpene lactone natural product, and 10 mg (25 μmol) of (guanidinium)$_2$(2,6-naphthaleneldisulfonate) (G_2NDS) apohost and afforded colorless block-shaped single crystals within 36 hours having the formula (G_2NDS)⊃((3aR)-(+)-Sclareolide) (**5**). NMR (400 MHz, *d*$_6$-DMSO): 8.02 (s, 2H), 7.80 (d, 2H), 7.61 (d, 2H), 6.83 (s, 12H), 2.4-0.85 (m, 26H). Single crystal X-ray diffraction determined that the compound crystallized in the $P2_12_12_1$ space group with the simple brick architecture. The Flack parameter, which reflects the correctness of chirality assignment, was found to be -0.08(4), indicating definitive determination of absolute configuration.

Crystallization of G_2NDS⊃(drospirenone)(methanol)$_{0.84}$(H_2O)$_{0.1}$ (6). Slow evaporation under ambient conditions of 1 mL of methanol containing 3 mg (8.2 μmol) of drospirenone and 3 mg (8.4 μmol) of (guanidinium)$_2$(2,6-naphthaleneldisulfonate) (G_2NDS) apohost afforded colorless block-shaped single crystals within 36 hours having the formula G_2NDS⊃(drospirenone)(methanol)$_{0.84}$(H_2O)$_{0.1}$ (**6**). NMR (400 MHz, d_6-DMSO): 8.10 (s, 2H), 7.90 (d, 2H), 7.69 (d, 2H), 6.94 (s, 12H), 3.17-3.15 (m, 2H), 2.10-1.95 (m, 5H), 1.23 (m, 12H), 1.04 (s, 3H)), 0.86 (s, 3H). Single crystal X-ray diffraction determined that the compound crystallized in the $P1$ space group with the zigzag brick architecture. The Flack parameter, which reflects the correctness of chirality assignment, was found to be 0.02(5), indicating definitive determination of absolute configuration.

Crystallization of G_2BPDS⊃(progesterone)(ethanol) (7a). Slow evaporation under ambient conditions of 2 mL of 1:1 (v/v) methanol:ethanol solution containing 5 mg (16 μmol) of progesterone and 23 mg (53 μmol) of (guanidinium)$_2$(4, 4'-biphenyldisulfonate) (G_2BPDS) apohost afforded block-shaped single crystals within 48 hours having the formula (G_2BPDS)⊃(progesterone)(ethanol) (**7a**). Crystals of neat progesterone formed concomitantly, precluding composition determination through NMR spectroscopy due to the difficulties separating the two kinds of crystals. Single crystal X-ray diffraction determined that the compound crystallized in the $P2_12_12_1$ space group with the simple brick architecture wherein two progesterone molecules lay back to back perpendicular to the BPDS pillars. The Flack parameter, which reflects the correctness of chirality assignment, was found to be 0.10(4), While this value is slightly larger than the other examples herein, this was attributable to the small size of the crystal examined and the associated low intensity of diffraction from the sample. Nonetheless, the measured Flack parameter is within the range required for confident assignment of the absolution configuration.

Crystallization of G_2BDPYDS⊃(progesterone) (7b). Slow evaporation under ambient conditions of a 2 mL 1:3 (v/v) ethanol:methanol solution containing 5 mg (16 μmol) of progesterone and 10 mg (18 μmol) of (guanidinium)$_2$(BDPYDS) (G_2BDPYDS) apohost afforded thin orange plates within 96 hours with the formula(G_2BDPYDS)⊃(progesterone) (**7b**). The proton NMR showed 1:1 stoichiometry of the G_2BDPY to progesterone, as can be demonstrated by studying the guanidinium peak as compared to a clear known methyl peak from the progesterone structure NMR (400 MHz, d_6-DMSO): 6.92 (s, 12H), 2.72-2.54 (m, 15H), 2.46-0.86 (m, 27H) 0.57 (s, 3H). Single crystal X-ray diffraction determined that the compound crystallized in the $P2_12_12_1$ space group with the simple brick architecture. The Flack parameter, which reflects the correctness of chirality assignment, was found to be 0.05(3) is within the range required for confident assignment of the absolution configuration.

Crystallization of G_2SDS⊃(7-acetyl-5,8-dihydroxy-4-isopropyl-1-methylbicyclo[4.3.0] nonane)$_{0.5}$(H_2O)$_{0.32}$ (8). Slow evaporation under ambient conditions of a 1 mL methanol solution containing 4 mg (16 μmol) of 7-acetyl-5,8-dihydroxy-4-isopropyl-1-methylbicyclo[4.3.0]nonane and 8 mg (17 μmol) of (guanidinium)$_2$(4,4'-stilbenedisulfonate) (G_2SDS) apohost afforded block-shaped single crystals within 72 hours with the formula G_2SDS⊃(7-acetyl-5,8-dihydroxy-4-isopropyl-1-methylbicyclo[4.3.0]nonane)$_{0.5}$(H_2O)$_{0.32}$ (**8**). Single crystals of guest-free G_2SDS formed concomitantly, precluding composition determination through NMR spectroscopy due to the difficulties separating the two kinds of crystals. Single crystal X-ray diffraction determined that the compound crystallized in the $P1$ space group with the crisscross bilayer architecture. The Flack parameter, which reflects the correctness of chirality assignment, was found to be 0.02(7), indicating definitive determination of absolute configuration.

Crystallization of G_3TSPHB⊃(neryl acetate)(isophorone)$_3$ (9). Slow evaporation under ambient conditions of 2 mL of methanol solution containing 50 μL (45 mg, 0.23 mmol) of neryl acetate, 150 μL (138 mg, 1 mmol) of isophorone and 10 mg (14 μmol) of (guanidinium)$_3$(tri(4-sulfophenyl)benzene) (G_3TSPHB) apohpost afforded needle-shaped single crystals within 36 hours with the formula (G_3TSPHB)⊃(neryl acetate)(isophorone)$_3$ (**9**). NMR (400 MHz, d_6-DMSO): 7.91 (s, 3H), 7.84 (d, 6H), 7.70 (d, 6H), 6.90 (s, 18H), 5.78 (s, 3H), 5.31 (t, 1H), 5.07 (t, 1H), 4.48 (d, 2H), 2.18 (s, 6H), 2.11 (s, 6H), 2.08 -1.98 (m, 7H), 1.90 (s, 9H), 1.71 (s, 3H), 1.64 (s, 3H), 1.56 (s, 3H), 0.96 (s, 18H). Single crystal X-ray diffraction determined that the compound crystallized in the $P2_12_12_1$ space group, in which the guests are confined in three crystallographically distinct channels. Pairs of isophorone molecules occupy two of the channels, which are flanked by two adjacent trisulfonate molecules. One additional equivalent of isophorone occupied the third channel. The isophorone molecules appear to anchor the neryl acetate, which is sandwiched by two TSPHB molecules thereby precluding disorder of the targeted guest.

Crystallization of $G_6(TSPB)_2$⊃(deacetylated pancuronium)$_2(H_2O)_{3.08}$ (10). Slow introduction of 1,4-dioxane vapor to a slightly acidic aqueous methanol solution (methanol was found to be the optimum solvent for this compound) at 40 °C containing 5 mg (6.8 μmol) of pancuronium bromide and 10 mg (10.6 μmol) of (guanidinium)$_4$(tetra(4-sulfophenyl)benzene) (G_4TSPB) apohost afforded block-shaped single crystals after seven days having the formula (G_6(1,1'-((2*S*,3*S*,5*S*,8*R*,9*S*,10*S*,13*S*,14*S*,16*S*,17*R*)-3,17-dihydroxy-10,13-dimethylhexadecahydro-1*H*-cyclopenta[*a*]phenanthrene-2,16-diyl)bis(1-methylpiperidin-1-ium))$_2$((TSPB)$_2$·(H_2O)$_{3.08}$) (a.k.a. ($G_6(TSPB)_2$⊃(deacetylated pancuronium)(H_2O)$_{3.08}$) (**10**). The crystals were embedded in the gel of an amorphous solid, which precluded their isolation and determination of their composition from NMR. Single crystal X-ray diffraction determined that the compound crystallized in the *C*2 space group with a non-traditional architecture. The crystal structure revealed that the guest was actually deacetylated pancuronium as a result of hydrolysis of the pancuronium acetyl groups during crystallization, demonstrating that reaction products could be captured in situ during crystallization of an inclusion compound. Furthermore, the X-ray data confirmed that the stereochemistry about the chiral centers was unaffected by hydrolysis as well as crystallization. The Flack parameter, which reflects the correctness of chirality assignment, was found to be 0.02(2).

Microscale crystallization of G_2NDS⊃((3aR)-(+)-Sclareolide) (5micro). Slow evaporation under ambient conditions of a 1 μL aliquot of a methanol solution in a plastic conical vial containing 5 μg (20 nmol) of (3aR)-(+)-Sclareolide and 8 μg (19.5 nmol) of (guanidinium)$_2$(2,6-naphthalenedisulfonate) (G_2NDS) apohost in a plastic conical vial afforded a single crystal of (G_2NDS)⊃((3aR)-(+)-Sclareolide). The aliquot was obtained from a parent methanol solution (6 mL) containing 30 mg of (3aR)-(+)-Sclareolide/azulene and 50 mg of G_2NDS dissolved in 6 mL methanol. Single crystal X-ray diffraction determined that the crystal structure was identical to that obtained for the larger-scale crystallization.

Supplementary Tables

Supplementary Table 1. Structural features of inclusion compound **1a** through **9**.

No.	Formula	Architecture	V_{pillar} (Å^3)[a]	nV_g (Å^3)[a]	nV_{sol} (Å^3)[a,b]	V_{inc} (Å^3)[a]	Overall packing fraction[c]	% V_{inc} occupied
**1a**	G_2BPDS⊃(guaiazulene)$_2$	Zigzag brick	226	402	0	780	66.2%	51.5%
**1b**	G_2ADS⊃(guaiazulene)$_2$	Zigzag brick	242	403	0	763	67.6%	52.8%
**2**	G_2NDS⊃(azulene)$_3$	Simple brick	200	369	0	662	69.9%	55.7%
**3**	G_2BPDS⊃(2,6-diisopropylaniline)$_2$	Zigzag brick	226	368	0	741	65.3%	49.7%
**4**	GBPMS⊃(S-(+)-carvone)	Continuously layered	187	151	0	325	67.9%	46.5%
**5**	G_2NDS⊃((3aR)-(+)-sclareolide)	Simple brick	199	244	0	558	63.7%	43.7%
**6**	G_2NDS⊃(drospirenone)(methanol)$_{0.84}$(H_2O)$_{0.1}$	Zizag brick	200	336	30	665	69.9%	55.0%
**7a**	G_2BPDS⊃(progesterone)(ethanol)	Simple brick	226	303	50	691	66.9%	51.1%
**7b**	G_2BDPYDS⊃(progesterone)	Simple brick	306	304	0	624	68.4%	48.7%
**8**	G_2SDS⊃(7-Acetyl-5,8-dihydroxy-4-isopropyl-1-methylbicyclo[4.3.0]nonane)$_{0.5}$(H_2O)$_{0.32}$	Crisscross bilayer	250	122	5	347	66.4%	36.6%[d]
**9**	G_2TSPHB⊃(neryl acetate)(isophorone)$_3$	Tricylindrical	402	201<br>420	0	1286	64.2%	48.3%

[a] Molecular volume (V_g, V_{pillar}, V_{inc}) calculations were performed using BIOVIA Materials Studio 2018. Connolly surface with a probe radius of zero is used for calculation of volumes of guest molecules (V_g) and organosulfonate pillars (V_{pillar}). Connolly surface with a 0.5 Å probe was used to calculate volume of inclusion cavities after removal of guest molecules and solvent molecules. All volumes are normalized to one formula unit.

[b] Volumes of each water, methanol and ethanol molecules used are 18 Å^3, 34 Å^3 and 50 Å^3, respectively.

[c] Overall packing fraction was calculated as the sum of volume of organosulfonate pillars, guest molecules and guanidinium cations (55 Å^3 per cation) in each unit cell divided by the volume of the unit cell.

[d] In the crisscross bilayer structure G_2SDS there is insufficient space for guest inclusion between the alternating pillars along the ribbon direction, resulting in unoccupied void space that reduces the overall %V_{inc}.

Supplementary Table 2. Selected crystallographic data.

**Compound name**	G_2BPDS⊃(guaiazulene)$_2$	G_2ADS⊃(guaiazulene)$_2$	G_2BDS⊃(azulene)$_3$	G_2BPDS⊃(2,6-diisopropylaniline)$_2$
**Structure file name**	**1a**	**1b**	**2**	**3**
**X-ray lab code**	17mdw14l	17mdw146l	17mdw25l	17mdw109l
**CCDC no.**	1867926	1867927	1867928	1867929
**Formula by X-ray**	$C_{44}H_{56}N_6O_6S_2$	$C_{46}H_{56}N_6O_6S_2$	$C_{42}H_{42}N_6O_6S_2$	$C_{38}H_{58}N_8O_6S_2$
**Formula weight**	829.06	853.08	790.93	787.04
**Crystal habit**	green plate	blue plate	blue plate	colorless needle
**Crystal size (mm)**	0.22 · 0.52 · 0.54	0.04 · 0.28 · 0.39	0.07 · 0.38 · 0.43	0.07 · 0.10 · 0.53
**Crystal system**	orthorhombic	orthorhombic	monoclinic	orthorhombic
**Space group (no.)**	*Pbca* (61)	*Pbca* (61)	$P2_1/n$ (14)	$P2_12_12_1$ (19)
a **(Å)**	13.8865(8)	13.1459(9)	7.6081(10)	12.5807(9)
b **(Å)**	12.9808(7)	12.7761(8)	21.400(3)	13.9645(11)
c **(Å)**	24.7551(14)	26.5707(17)	11.9288(16)	24.5444(18)
α **(°)**	90	90	90	90
β **(°)**	90	90	90.752(2)	90
γ **(°)**	90	90	90	90
V **(Å^3)**	4462.3(4)	4462.6(5)	1942.0(4)	4312.0(6)
Z	4	4	2	4
D_c **(g cm^{-3})**	1.234	1.270	1.353	1.212
F**(000)**	1768	1816	832	1688
μ **(mm^{-1})**	0.172	0.174	0.194	0.175
**Total reflections**	66096	34785	22656	67760
**Unique reflections**	5552	3209	3327	10716
R_{int}	0.0409	0.0709	0.0593	0.0193
R_1[a] $[I > 2\sigma(I)]$	0.0575	0.0423	0.0410	0.0302
wR_2[b] **(all data)**	0.1599	0.1154	0.1006	0.0843
**GOF (all data)**	1.022	1.031	1.048	1.037
**Flack parameter**	n.a.[c]	n.a.	n.a.	0.49(5)

[a] $R_1 = \Sigma||F_o|-|F_c||/\Sigma|F_o|$; [b] $wR_2=\{\Sigma[w(F_o^2-F_c^2)^2]/\Sigma[w(F_o^2)^2]\}^{1/2}$; [c] n.a. – not applicable.

Supplementary Table 2 (continued). Selected crystallographic data.

**Compound name**	GBPMS⊃(S-(+)-carvone)	G_2NDS⊃((3aR)-(+)-sclareolide)	G_2NDS⊃(drospirenone)·(methanol)$_{0.84}$(H_2O)$_{0.1}$	G_2BPDS⊃(progesterone)(ethanol)
**Structure file name**	**4**	**5**	**6**	**7a**
**X-ray lab code**	17mdw122l	17mdw22l	17mdw129l	17mdw18l
**CCDC no.**	1867930	1867931	1867932	1867933
**Formula by X-ray**	$C_{23}H_{29}N_3O_4S$	$C_{28}H_{44}N_6O_8S_2$	$C_{36.84}H_{51.37}N_6O_{9.94}S_2$	$C_{37}H_{56}N_6O_9S_2$
**Formula weight**	443.55	656.81	801.52	792.99
**Crystal habit**	colorless prism	colorless plate	colorless plate	colorless rod
**Crystal size (mm)**	0.16 · 0.22 · 0.54	0.07 · 0.17 · 0.56	0.03 · 0.16 · 0.26	0.11 · 0.14 · 0.33
**Crystal system**	orthorhombic	orthorhombic	triclinic	Orthorhombic
**Space group (no.)**	$P2_12_12_1$ (19)	$P2_12_12_1$ (19)	$P1$ (1)	$P2_12_12_1$ (1)
***a* (Å)**	7.5057(5)	12.8742(12)	12.126(3)	11.277(2)
***b* (Å)**	12.0567(8)	14.5355(14)	12.492(3)	26.267(5)
***c* (Å)**	25.5858(17)	18.5423(18)	14.259(3)	27.802(5)
α (°)	90	90	92.657(4)	90
β (°)	90	90	91.256(4)	90
γ (°)	90	90	116.190(4)	90
V (Å^3)	2315.4(3)	3469.9(6)	1934.0(8)	8235(2)
Z	4	4	2	8
D_c (g cm^{-3})	1.272	1.257	1.376	1.279
F(000)	944	1400	852	3392
μ (mm^{-1})	0.173	0.206	0.203	0.188
**Total reflections**	29701	47188	33982	52366
**Unique reflections**	5720	7149	15354	8700
R_{int}	0.0389	0.0649	0.0542	0.0669
R_1[a] [$I > 2\sigma(I)$]	0.0333	0.0448	0.0640	0.0797
wR_2[b] (all data)	0.0880	0.1168	0.1568	0.1882
**GOF (all data)**	1.050	1.030	0.997	1.124
**Flack parameter**	0.02(3)	-0.08(4)	0.02(5)	0.10(4)

[a] $R_1 = \Sigma||F_o|-|F_c||/\Sigma|F_o|$; [b] $wR_2=\{\Sigma[w(F_o^2-F_c^2)^2]/\Sigma[w(F_o^2)^2]\}^{1/2}$; [c] n.a. – not applicable.

Supplementary Table 2 (continued). Selected crystallographic data.

**Compound name**	G_2BDPYDS⊃(progesterone)	G_2SDS⊃(7-Acetyl-5,8-dihydroxy-4-isopropyl-1-methylbicyclo[4.3.0] nonane)$_{0.5}$(H_2O)$_{0.32}$	G_2TSPHB⊃(neryl acetate)(isophorone)$_3$	$G_{5.33}$(TSPB)$_2$⊃(deacetylated pancuronium)(H_2O)$_{3.08}$
**Structure file name**	**7b**	**8**	**9**	**10**
**X-ray lab code**	19mdwll	17mdw150l	17mdw32l	17mdw113l
**CCDC no.**	1905538	1867934	1867935	1867936
**Formula by X-ray**	$C_{37}H_{57}B_1F_2N_8O_8S_2$	$C_{23.5}H_{35.35}N_6O_{7.82}S_2$	$C_{66}H_{95}N_9O_{14}S_3$	$C_{48.16}H_{65.07}N_{8.99}O_{14.54}S_2$
**Formula weight**	854.83	591.12	1334.68	1130.91
**Crystal habit**	yellow plate	colorless prism	colorless rod	colorless block
**Crystal size (mm)**	0.08 · 0.17 · 0.36	0.05 · 0.10 · 0.13	0.18 · 0.22 · 0.52	0.18 · 0.20 · 0.44
**Crystal system**	orthorhombic	triclinic	orthorhombic	monoclinic
**Space group (no.)**	$P2_12_12_1$ (19)	$P1$ (1)	$P2_12_12_1$ (19)	$C2$ (5)
***a* (Å)**	9.496(2)	7.6082(16)	7.5738(5)	36.027(2)
***b* (Å)**	14.992(4)	12.009(3)	24.6695(15)	19.8273(13)
***c* (Å)**	29.553(7)	31.990(7)	39.618(2)	18.7832(12)
α (°)	90	86.999(4)	90	90
β (°)	90	86.382(4	90	117.7178(10)
γ (°)	90	89.984(4)	90	90
***V* (Å^3)**	4207.4(18)	2913.0(10)	7402.3(8)	11877.4(13)
Z	4	4	4	8
**D_c (g cm^{-3})**	1.350	1.348	1.198	1.265
***F*(000)**	1816	1252	2856	4779
**μ (mm^{-1})**	0.195	0.237	0.165	0.227
**Total reflections**	47379	30758	90056	73146
**Unique reflections**	7474	16861	15179	21054
**R_{int}**	0.0613	0.0547	0.0569	0.0398
**R_1[a] [$I > 2\sigma(I)$]**	0.0519	0.0626	0.0563	0.0771
**wR_2[b] (all data)**	0.1215	0.1595	0.1344	0.2411
**GOF (all data)**	1.087	0.988	1.125	1.053
**Flack parameter**	0.05(3)	0.02(7)	0.45(8)	0.02(2)

[a]$R_1 = \Sigma||F_o|-|F_c||/\Sigma|F_o|$; [b]$wR_2=\{\Sigma[w(F_o^2-F_c^2)^2]/\Sigma[w(F_o^2)^2]\}^{1/2}$; [c]n.a. – not applicable.

Supplementary Table 2 (continued). Selected crystallographic data for G_2NDS·((3aR)-(+)-Sclareolide) **(5micro)**.

**Compound name**	G_2NDS⊃((3aR)-(+)-sclareolide)
**Structure file name**	**5micro**
**X-ray lab code**	17mdw128l
**CCDC no.**	1867937
**Formula by X-ray**	$C_{28}H_{44}N_6O_8S_2$
**Formula weight**	656.81
**Crystal habit**	colorless prism
**Crystal size (mm)**	0.05 · 0.09 · 0.12
**Crystal system**	orthorhombic
**Space group (no.)**	$P2_12_12_1$ (19)
a **(Å)**	12.851(5)
b **(Å)**	14.549(5)
c **(Å)**	18.536(7)
α (°)	90
β (°)	90
γ (°)	90
V (Å^3)	3466(2)
Z	4
D_c (g cm^{-3})	1.259
F(000)	1400
μ (mm^{-1})	0.207
**Total reflections**	44879
**Unique reflections**	7135
R_{int}	0.1618
R_1[a] [$I > 2 ʃ (I)$]	0.0690
wR_2[b] (all data)	0.1617
**GOF (all data)**	1.015
**Flack parameter**	0.02(12)

[a]$R_1 = \Sigma||F_o|-|F_c||/\Sigma|F_o|$; [b]$wR_2=\{\Sigma[w(F_o^2-F_c^2)^2]/\Sigma[w(F_o^2)^2]\}^{1/2}$; [c]n.a. – not applicable.

Supplementary Table 3. Comparison of the structure refinement for guaiazulene and 2,6-diisopropylaniline using the single-step crystallization GS framework method with crystalline sponge method (CSM) and improved CSM.[9]

Compound	Method	R_1	wR_2	Number of restraints	Occupancy	Squeeze? Y/N
Guaiazulene	GS	0.0424	0.1059	0	100%	No
	CSM	0.0859	0.3021	71	~60%	Yes
	CSM improved	0.0379	0.1035	0	100%	No
2,6-diisopropyl-aniline	GS	0.0302	0.0829	0	100%	No
	CSM	0.1182	0.3520	64	~75%	Yes
	CSM improved	0.0653	0.1541	78	100%	No

Supplementary Table 4. Bond lengths of guaiazulene determined by the crystalline sponge method (CSM) and in compounds **1a** and **1b**.

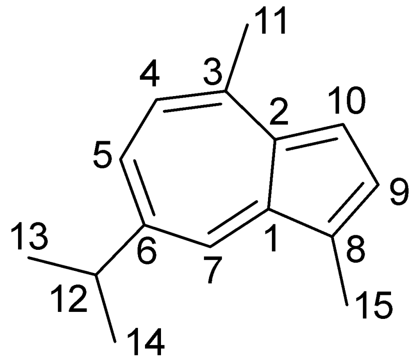

Bond length (Å)	CSM Nature (2012)	CSM IUCrJ (2016)	**1a***	**1b***
C1-C2	1.505(10)	1.458(11)	1.484(5)	1.501(4)
C2-C3	1.381(9)	1.399(10)	1.393(5)	1.383(4)
C3-C4	1.393(7)	1.360(10)	1.382(6)	1.414(5)
C4-C5	1.410(9)	1.394(10)	1.402(6)	1.388(4)
C5-C6	1.402(9)	1.348(10)	1.410(6)	1.404(5)
C6-C7	1.397(9)	1.430(10)	1.409(6)	1.386(5)
C7-C1	1.378(10)	1.416(10)	1.393(5)	1.381(4)
C1-C8	1.391(10)	1.412(10)	1.418(5)	1.416(4)
C8-C9	1.393(10)	1.405(13)	1.382(6)	1.396(4)
C9-C10	1.389(10)	1.407(13)	1.395(6)	1.382(5)
C10-C2	1.361(10)	1.396(10)	1.402(6)	1.401(5)
C3-C11	1.490(10)	1.542(10)	1.500(6)	1.515(4)
C6-C12	1.492(10)	1.520(10)	1.504(8)	1.528(4)
C12-C13	1.497(10)	1.574(14)	1.525(9)	1.525(5)
C12-C14	1.490(10)	1.512(11)	1.511(9)	1.520(5)
C8-C15	1.492(10)	1.511(14)	1.490(6)	1.494(4)

*The bond lengths of the major disordered component are listed.

Supplementary Table 4. Bond lengths of 2,6-diisopropylaniline determined by the crystalline sponge method (CSM) and in compound **3**

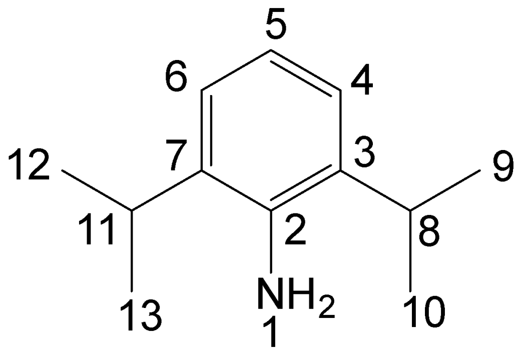

Bond length (Å)	CSM Nature (2012)	CSM IUCrJ (2016)	**3** molecule 1*	**3** molecule 2*
N1-C2	1.371(9)	1.384(16)	1.404(2)	1.399(2)
C2-C3	1.393(9)	1.42(2)	1.416(3)	1.413(3)
C3-C4	1.393(9)	1.37(2)	1.390(3)	1.392(3)
C4-C5	1.386(9)	1.37(2)	1.389(3)	1.389(3)
C5-C6	1.389(9)	1.34(2)	1.386(3)	1.387(3)
C6-C7	1.395(9)	1.440(18)	1.391(3)	1.396(3)
C7-C2	1.394(9)	1.389(18)	1.408(3)	1.412(3)
C3-C8	1.54(3)	1.50(2)	1.522(3)	1.528(3)
C8-C9	1.533(17)	1.45(2)	1.531(3)	1.529(3)
C8-C10	1.547(18)	1.34(3)	1.539(3)	1.541(3)
C7-C11	1.51(3)	1.46(2)	1.524(3)	1.525(3)
C11-C12	1.595(17)	1.55(2)	1.531(3)	1.529(3)
C11-C13	1.522(9)	1.56(6)	1.535(3)	1.532(3)

*The asymmetric unit contains two 2,6-diisopropylaniline molecules.

Supplementary Table 5. Single crystal data for guest molecules discussed in this article that have been reported previously. These do not include molecular structures solved using the crystalline sponge method.

**Compound name**	Azulene	Azulene	(S)-(+)-Carvone ·(para-hexanoyl calix[4]arene)	catena-((m-(benzene-1,3,5-triyl)tris(methylene) tris(pyridine-4-carboxylate))-silver trifluoromethanesulfonate (S)-(+)-carvone solvate)
**CCDC no.**	1833585[10]	1104286[11]	296183[12]	1556271[13]
**Crystal system**	monoclinic	monoclinic	monoclinic	triclinic
**Space group (no.)**	$P2_1/a$	$P2_1/a$	$P2_1$	*P-1*
***a* (Å)**	7.7154(2)	7.884	15.371(3)	9.0164(2)
***b* (Å)**	5.9019(1)	5.988	21.945(4)	13.2877(2)
***c* (Å)**	7.6969(2)	7.840	15.966(2)	15.6254(3)
**α (°)**	90	90	90	108.1750(10)
**β (°)**	100.411(2)	101.55	90.07(3)	95.0300(10)
**γ (°)**	90	90	90	103.7260(10)
**V (Å^3)**	344.71(1)	362.627	5385.6(19)	1701.12(6)
**Z**	2	2	4	1
**D_c (g cm^{-3})**	1.235	1.174	1.331	1.596
**F(000)**	136.0	N/A	2344	830
**ρ (mm^{-1})**	0.07	N/A	0.086	0.730
**Total reflections**	35883	N/A	N/A	24565
**Unique reflections**	3834	N/A	8434	6660
**R_{int}**	0.038	N/A	N/A	0.0329
**R_1^a [$I > 2 ʃ (I)$]**	0.043	0.13	0.0874	0.0792
**wR_2^b (all data)**	0.1215	N/A	0.2301	0.2286
**GOF (all data)**	1.087	N/A	1.061	1.053
**Flack parameter**	N/A	N/A	0(2)	N/A
**Radiation wavelength (Å)**	0.71073	1.5406	0.9726	0.71073
**Temperature (K)**	100	N/A	173(2)	173(2)
**Stand alone?**	Y	Y	N	N
**Year reported**	2018	1962	2006	2017

$^aR_1 = \Sigma||F_o|-|F_c||/\Sigma|F_o|$; $^bwR_2=\{\Sigma[w(F_o^2-F_c^2)^2]/\Sigma[w(F_o^2)^2]\}^{1/2}$; ^{c}n.a. – not applicable.

Supplementary Table 5 (continued). Single crystal data for guest molecules discussed in this article that have been reported previously. These do not include molecular structures solved using the crystalline sponge method.

**Compound name**	5,11,17,23-p-Tetra(3-cyclohexyl)propanoyl-25,26,27,28-tetrahydroxycalix[4]arene (+)-carvone clathrate	Drospirenone	Drospirenone	Drospirenone
**CCDC no.**	780767[14]	620693[15]	903900[16]	1489513[17]
**Crystal system**	monoclinic	orthorhombic	orthorhombic	orthorhombic
**Space group (no.)**	$P2_1$	$P2_12_12_1$	$P2_12_12_1$	$P2_12_12_1$
***a* (Å)**	15.9123(11)	6.1020(17)	12.236(2)	12.2379(5)
***b* (Å)**	24.6729(16)	14.450(3)	12.592(2)	12.6069(8)
***c* (Å)**	16.1465(10)	23.763(8)	12.879(2)	12.8921(8)
α **(°)**	90	90	90	90
β **(°)**	90.295(4)	90	90	90
γ **(°)**	90	90	90	90
***V* (Å^3)**	6339.1(7)	2095.3(10)	1984.2(5)	1989.01(19)
Z	4	4	4	4
**D_c (g cm^{-3})**	1.181	1.270	1.227	1.224
***F*(000)**	2440	864	792	792
**ρ (mm^{-1})**	0.076	0.088	0.079	0.079
**Total reflections**	122207	2301	8188	5761
**Unique reflections**	12480	2190	4164	3794
**R_{int}**	0.0586	0.0230	0.0357	0.0282
**R_1[a] [$I > 2\sigma(I)$]**	0.0713	0.0516	0.0561	0.0522
**wR_2[b] (all data)**	0.2116	0.1244	0.1138	0.1013
**GOF (all data)**	1.091	1.064	1.054	1.019
**Flack parameter**	N/A	N/A	1.3(19)	-3(2)
**Radiation wavelength (Å)**	0.71073	0.71073	0.71073	0.71073
**Temperature (K)**	193(2)	293(2)	293(2)	293(2)
**Stand alone?**	N	Y	Y	Y
**Year reported**	2010	2006	2012	2016

[a]$R_1 = \Sigma||F_o|-|F_c||/\Sigma|F_o|$; [b]$wR_2=\{\Sigma[w(F_o^2-F_c^2)^2]/\Sigma[w(F_o^2)^2]\}^{1/2}$; [c]n.a. – not applicable.

Supplementary Table 5 (continued). Single crystal data for guest molecules discussed in this article that have been reported previously. These do not include molecular structures solved using the crystalline sponge method.

**Compound name**	Drospirenone	(3aR)-(+)-sclareolide	Progesterone	Progesterone
**CCDC no.**	943279[18]	1436060[19]	1238100[20]	1238097[21]
**Crystal system**	monoclinic	monoclinic	orthorhombic	orthorhombic
**Space group (no.)**	$P2_1$	$P2_1$	$P2_12_12_1$	$P2_12_12_1$
a **(Å)**	6.9415(9)	7.4988(5)	12.559(2)	6.252(2)
b **(Å)**	13.2918(17)	10.6861(7)	13.798(2)	12.592(3)
c **(Å)**	10.6904(14)	9.4162(7)	10.340(2)	22.488(2)
α **(°)**	90	90	90	90
β **(°)**	96.511(2)	110.507(3)	90	90
γ **(°)**	90	90	90	90
V **(Å³)**	980.0(2)	706.73(8)	1791.81	1770.37
Z	2	2	4	4
D_c **(g cm⁻³)**	1.242	1.177	1.166	1.18
***F*(000)**	396	276	N/A	N/A
ρ **(mm⁻¹)**	0.080	0.075	N/A	N/A
**Total reflections**	5075	6262	N/A	N/A
**Unique reflections**	1812	2329	N/A	N/A
R_{int}	0.0162	0.0307	N/A	N/A
R_1[a] **[$I > 2\sigma(I)$]**	0.0357	0.0340	0.047	0.123
wR_2[b] **(all data)**	0.0873	0.0771	N/A	N/A
**GOF (all data)**	1.487	1.056	N/A	N/A
**Flack parameter**	0(10)	-1.5(12)	N/A	N/A
**Radiation wavelength (Å)**	0.71073	0.71073	0.71073	0.71073
**Temperature (K)**	273(2)	100(2)	295	295
**Stand alone?**	Y	Y	Y	Y
**Year reported**	2013	2015	1972	1975

[a]$R_1 = \Sigma||F_o|-|F_c||/\Sigma|F_o|$; [b]$wR_2=\{\Sigma[w(F_o^2-F_c^2)^2]/\Sigma[w(F_o^2)^2]\}^{1/2}$; [c]n.a. – not applicable.

Supplementary Table 5 (continued). Single crystal data for guest molecules discussed in this article that have been reported previously. These do not include molecular structures solved using the crystalline sponge method.

**Compound name**	Progesterone·(4-bromophenol)	7-Acetyl-5,8-dihydroxy-4-isopropyl-1-methylbicyclo[4.3.0] nonane·0.5(toluene)	Pancuronium dibromide dichloromethane solvate monohydrate
**CCDC no.**	753857[22]	179908[23]	1227865[24]
**Crystal system**	monoclinic	orthorhombic	orthorhombic
**Space group (no.)**	$P2_1$	$P4_12_12_1$	$P2_12_12_1$
a **(Å)**	7.1675(2)	14.495(2)	11.10(3)
b **(Å)**	17.1472(5)	14.495(2)	13.99(4)
c **(Å)**	19.5212(8)	17.365(3)	26.07(6)
α **(°)**	90	90	90
β **(°)**	90.3820(10)	90	90
γ **(°)**	90	90	90
V **(Å^3)**	2399.15(14)	3648.5(10)	4048.38
Z	4	8	4
D_c **(g cm^{-3})**	1.350	1.094	1.37099
F**(000)**	1024	1320	N/A
ρ **(mm^{-1})**	0.080	N/A	N/A
**Total reflections**	25116	N/A	N/A
**Unique reflections**	13170	3758	N/A
R_{int}	0.0549	0.0278	N/A
R_1[a] **[$I > 2 \sigma(I)$]**	0.0589	0.0466	N/A
wR_2[b] **(all data)**	0.1115	0.1433	N/A
**GOF (all data)**	1.126		N/A
**Flack parameter**	0.017(11)	0.03(26)	N/A
**Radiation wavelength (Å)**	0.71073	1.5406	N/A
**Temperature (K)**	120(2)	293(2)	295
**Stand alone?**	N	N	N
**Year reported**	2010	2002	1971

[a]$R_1 = \Sigma||F_o|-|F_c||/\Sigma|F_o|$; [b]$wR_2=\{\Sigma[w(F_o^2-F_c^2)^2]/\Sigma[w(F_o^2)^2]\}^{1/2}$; [c]n.a. – not applicable.

Supplementary Figures

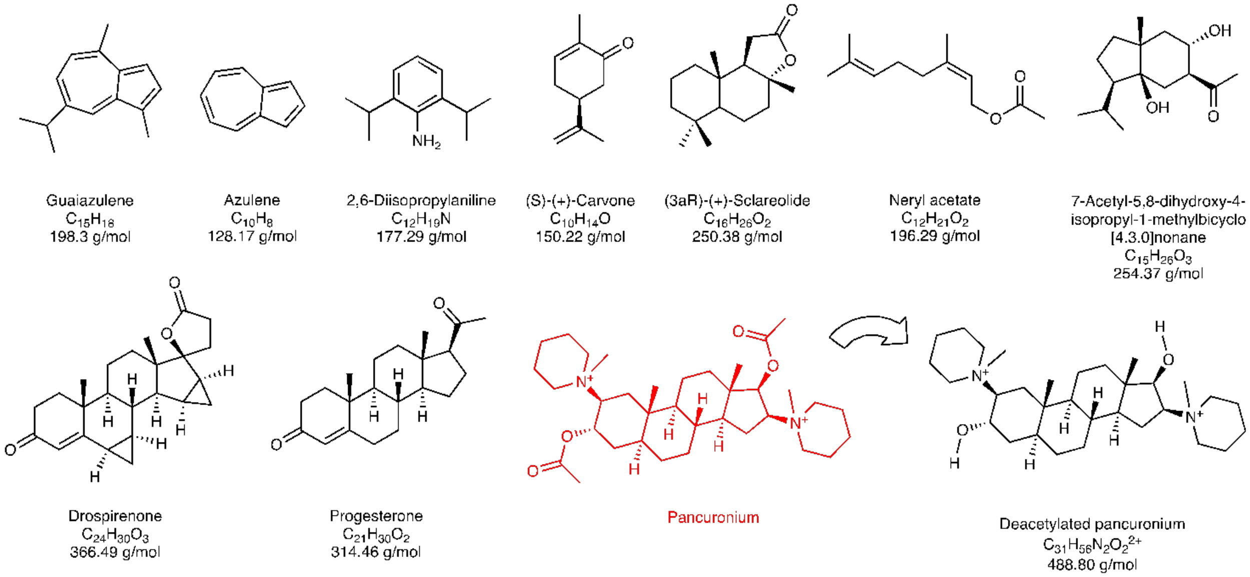

Supplementary Figure 1. Target molecules included in GS frameworks and characterized by single crystal X-ray diffraction.

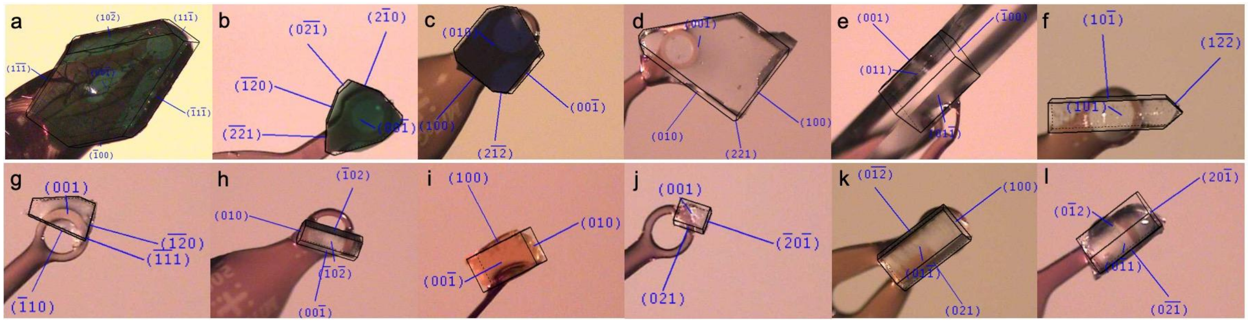

Supplementary Figure 2. Crystal images and Miller indices of largest faces of (a) $(G_2BPDS)\supset(\text{guaiazulene})_2$ (**1a**), (b) $(G_2ADS)\supset(\text{guaiazulene})_2$ (**1b**), (c) $(G_2NDS)\supset(\text{azulene})_3$ (**2**), (d) $(G_2BPDS)\supset(\text{2,6-diisopropylaniline})_2$ (**3**), (e) (GBPMS)⊃(S-(+)-Carvone) (**4**), (f) (G_2NDS)⊃((3aR)-(+)-Sclareolide) (**5**), (g) $(G_2NDS)\supset(\text{drospirenone})(\text{methanol})_{0.84}(H_2O)_{0.1}$ (**6**), (h) (G_2BPDS)⊃(Progesterone)(ethanol) (**7a**), (i) $(G_2BDPYDS)$⊃(Progesterone) (**7b**), (j) $(G_2SDS)\supset(\text{7-Acetyl-5,8-dihydroxy-4-isopropyl-1-methylbicyclo[4.3.0]nonane})_{0.5}(H_2O)_{0.32}$ (**8**), (k) $(G_3TSPHB)\supset(\text{neryl acetate})(\text{isophorone})_3$ (**9**), (l) $G_6(TSPB)_2\supset(\text{deacetylated pancuronium})_2(H_2O)_{3.08}$ (**10**).

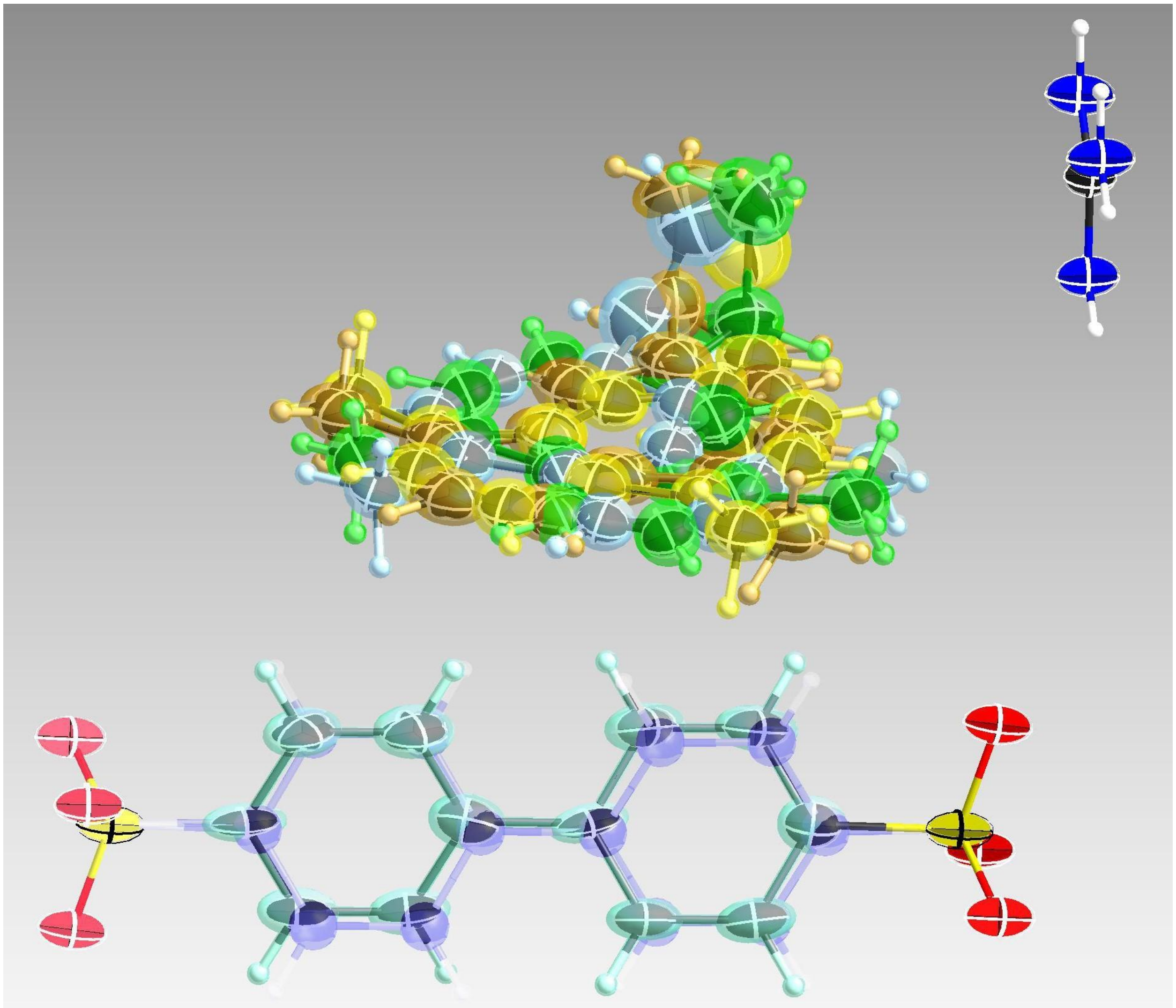

Supplementary Figure 3. Molecular structure of **1a** depicted as ellipsoids in 50% probability.

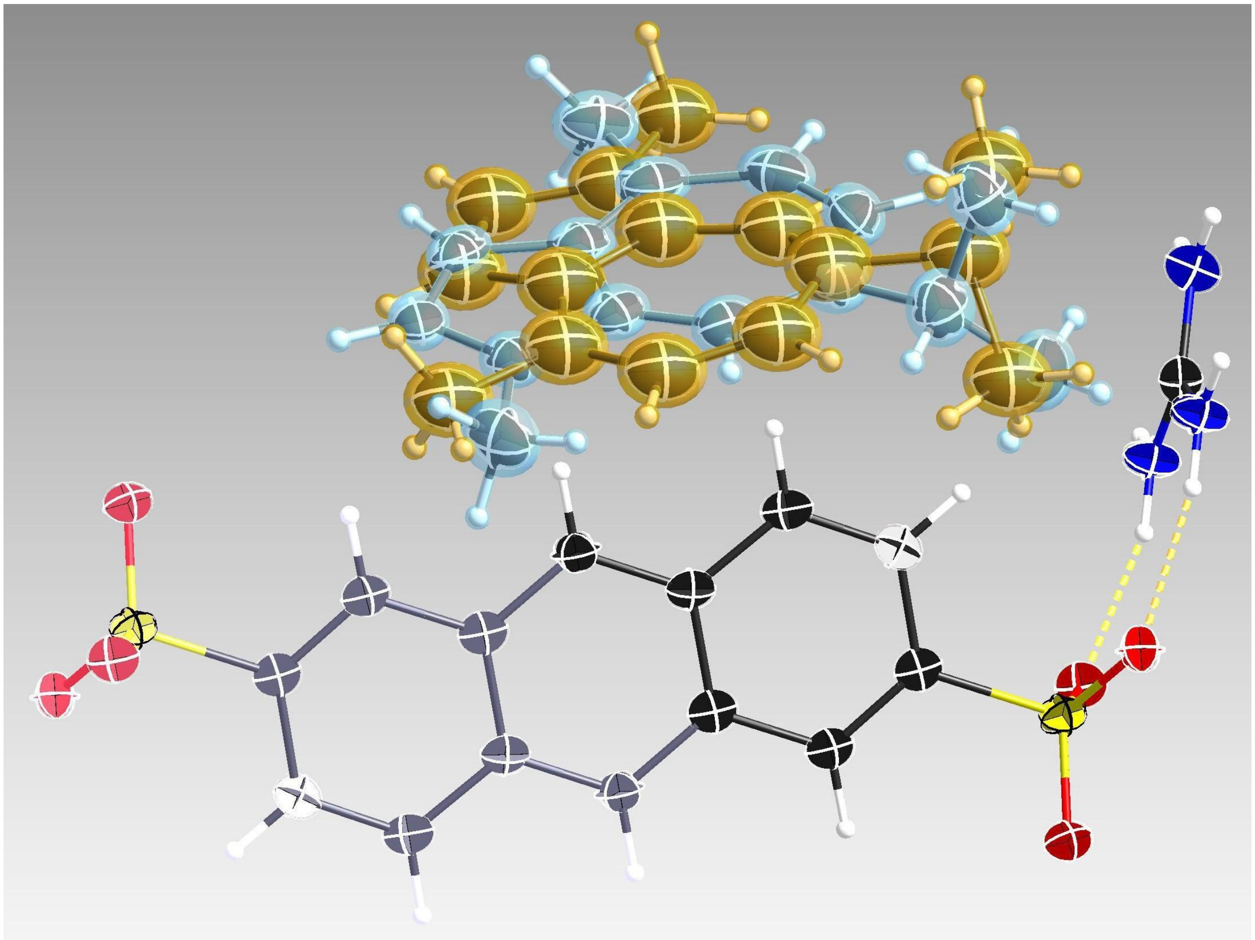

Supplementary Figure 4. Molecular structure of **1b** depicted as ellipsoids in 50% probability.

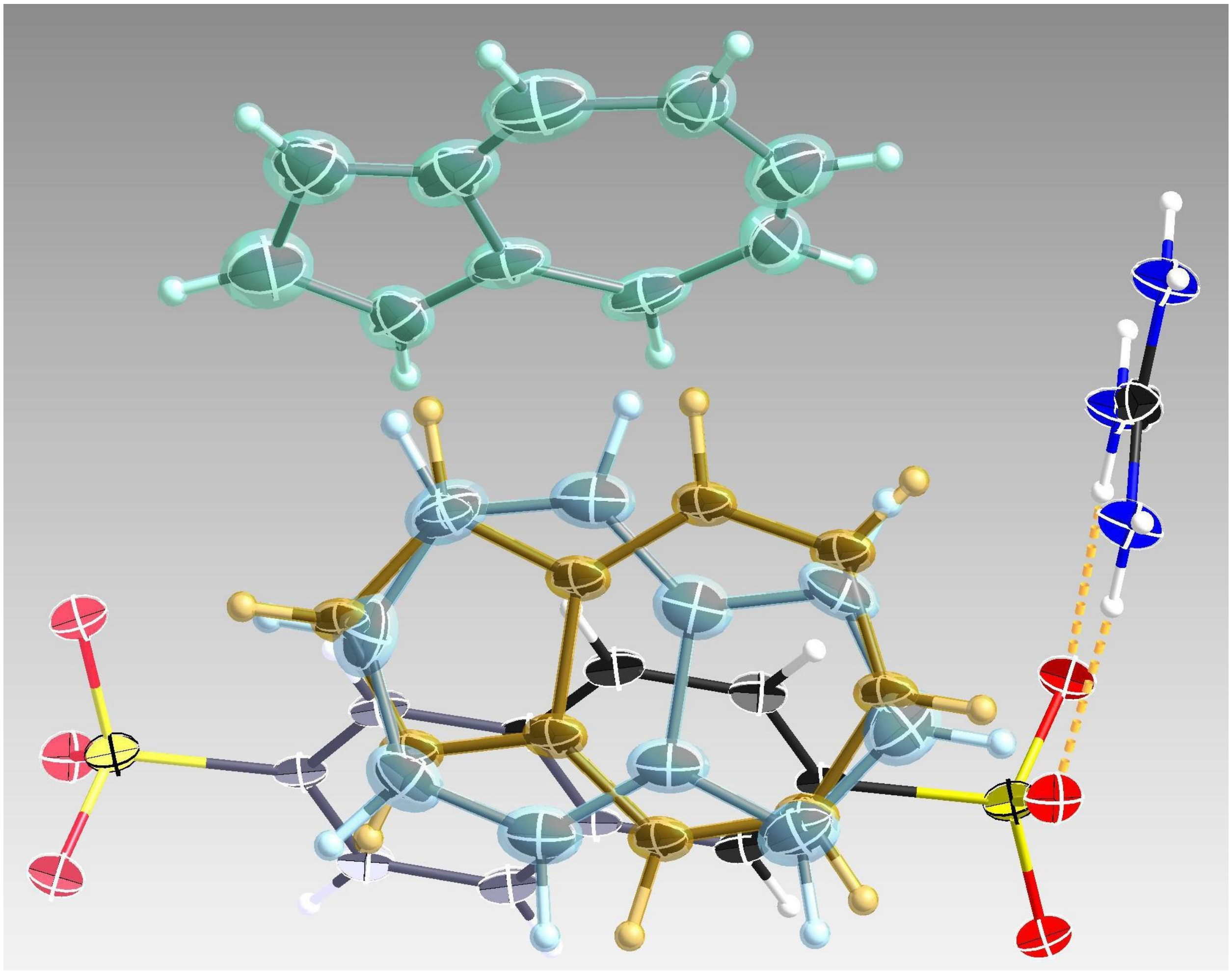

Supplementary Figure 5. Molecular structure of **2** depicted as ellipsoids in 50% probability.

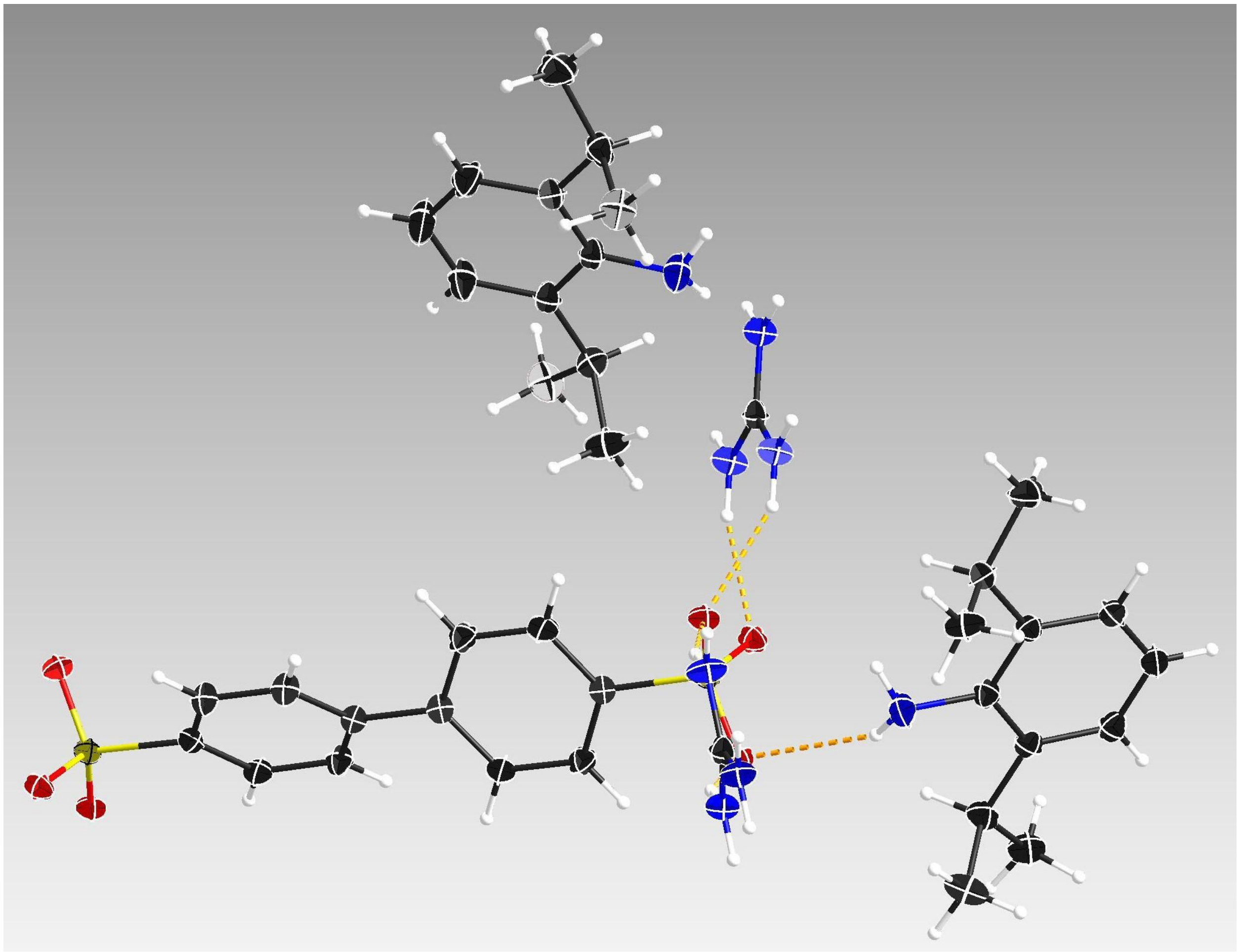

Supplementary Figure 6. Molecular structure of **3** depicted as ellipsoids in 50% probability.

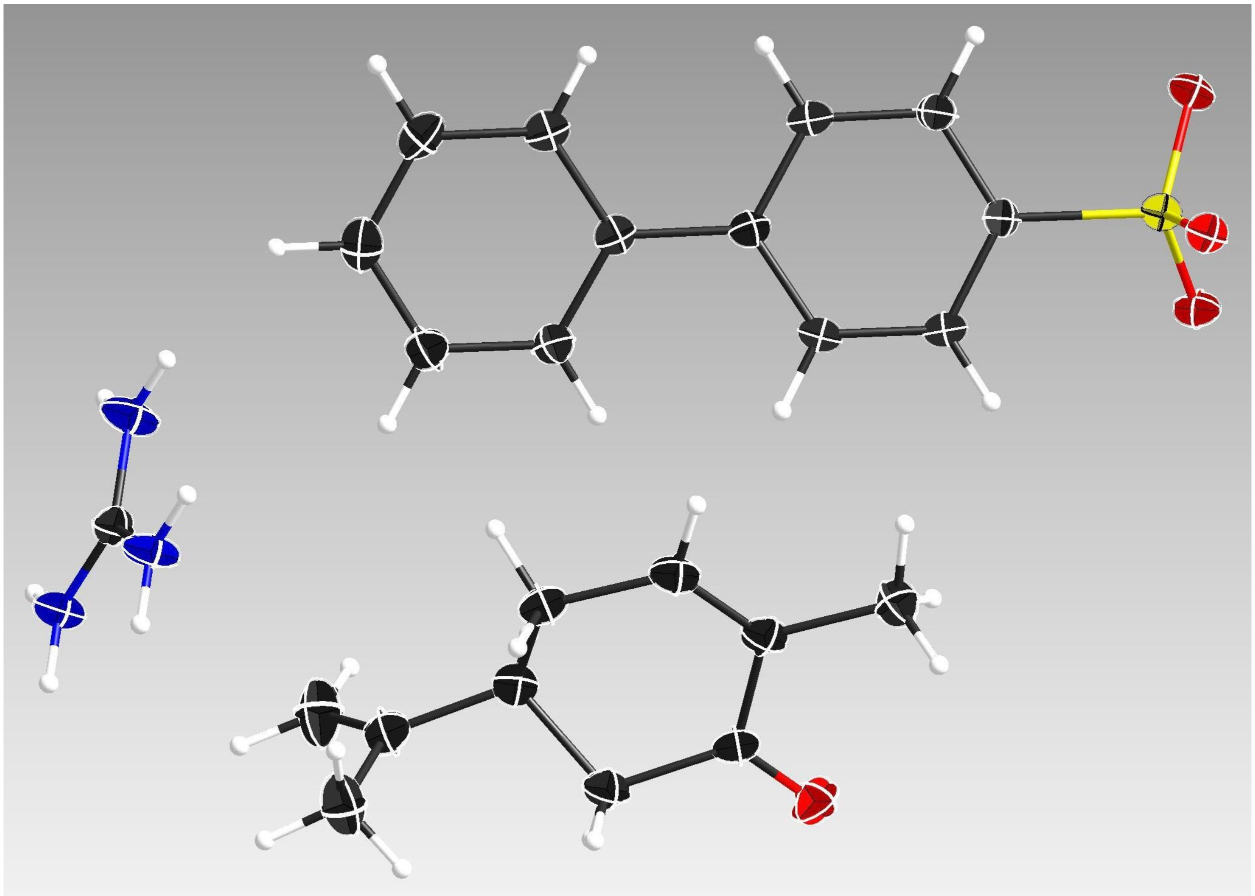

Supplementary Figure 7. Molecular structure of **4** depicted as ellipsoids in 50% probability.

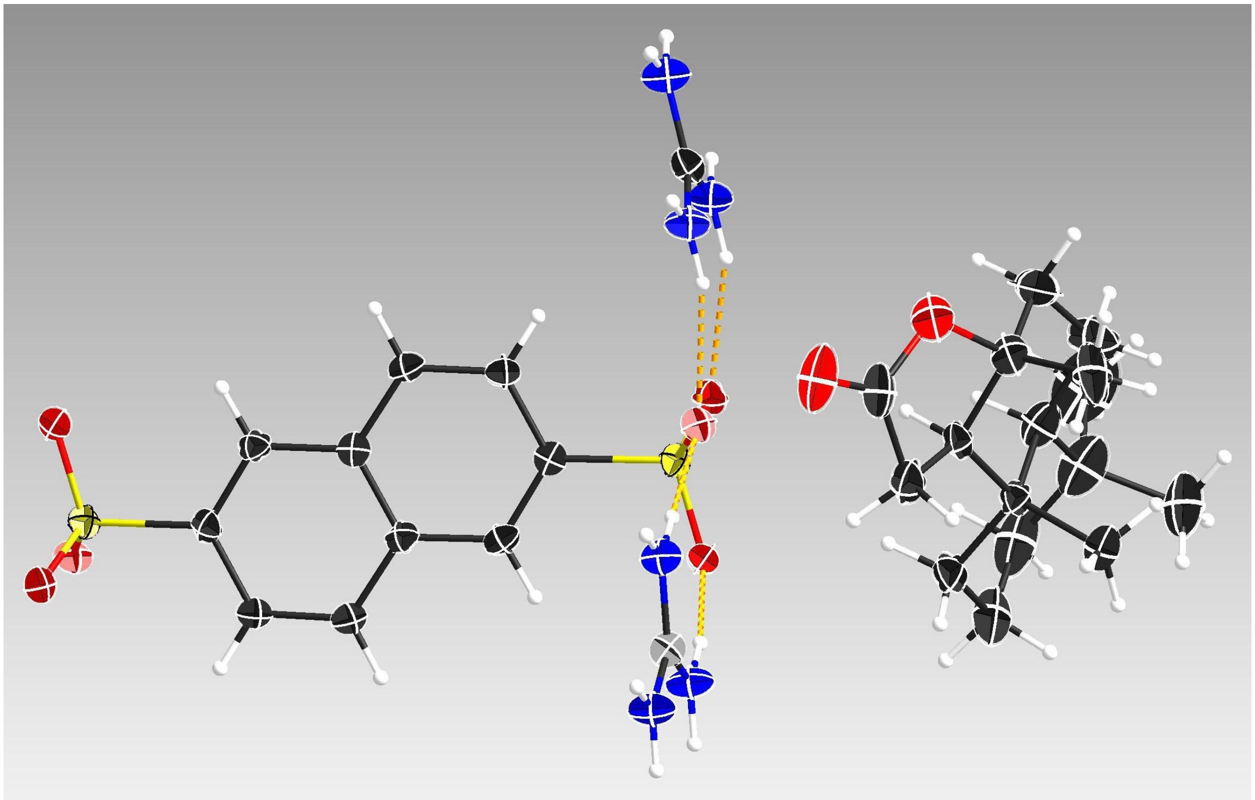

Supplementary Figure 8. Molecular structure of **5** depicted as ellipsoids in 50% probability.

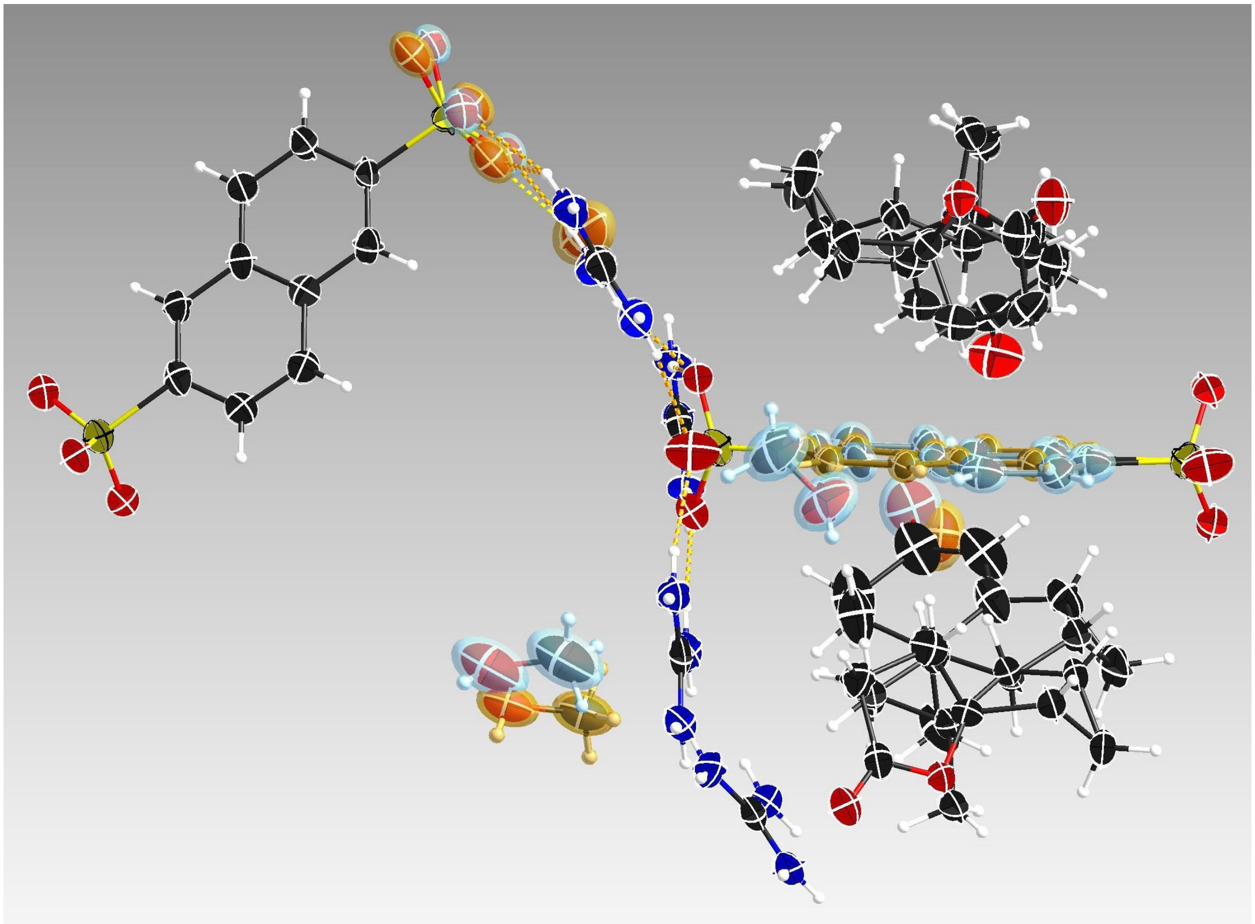

Supplementary Figure 9. Molecular structure of **6** depicted as ellipsoids in 50% probability.

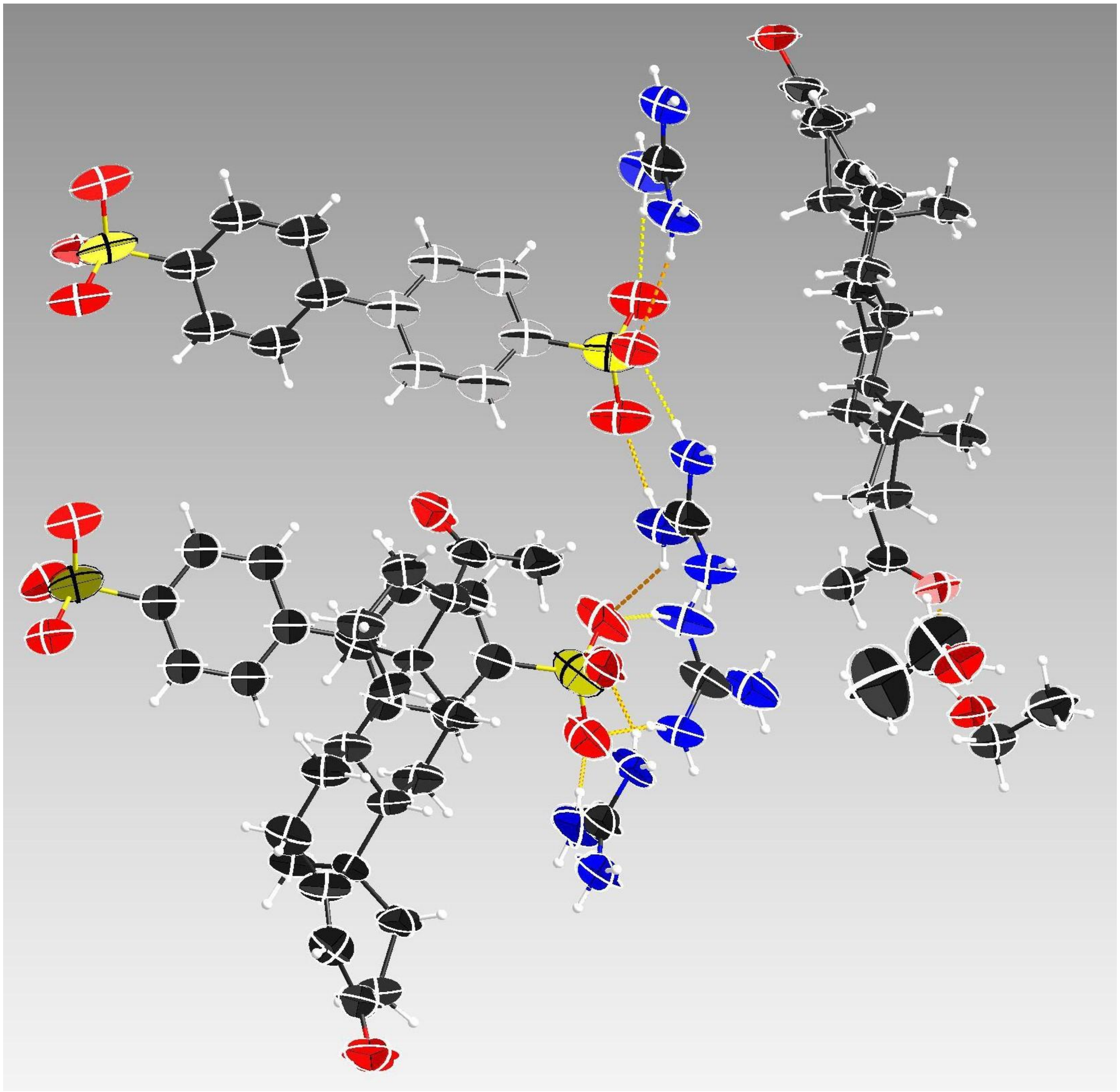

Supplementary Figure 10. Molecular structure of **7a** depicted as ellipsoids in 50% probability.

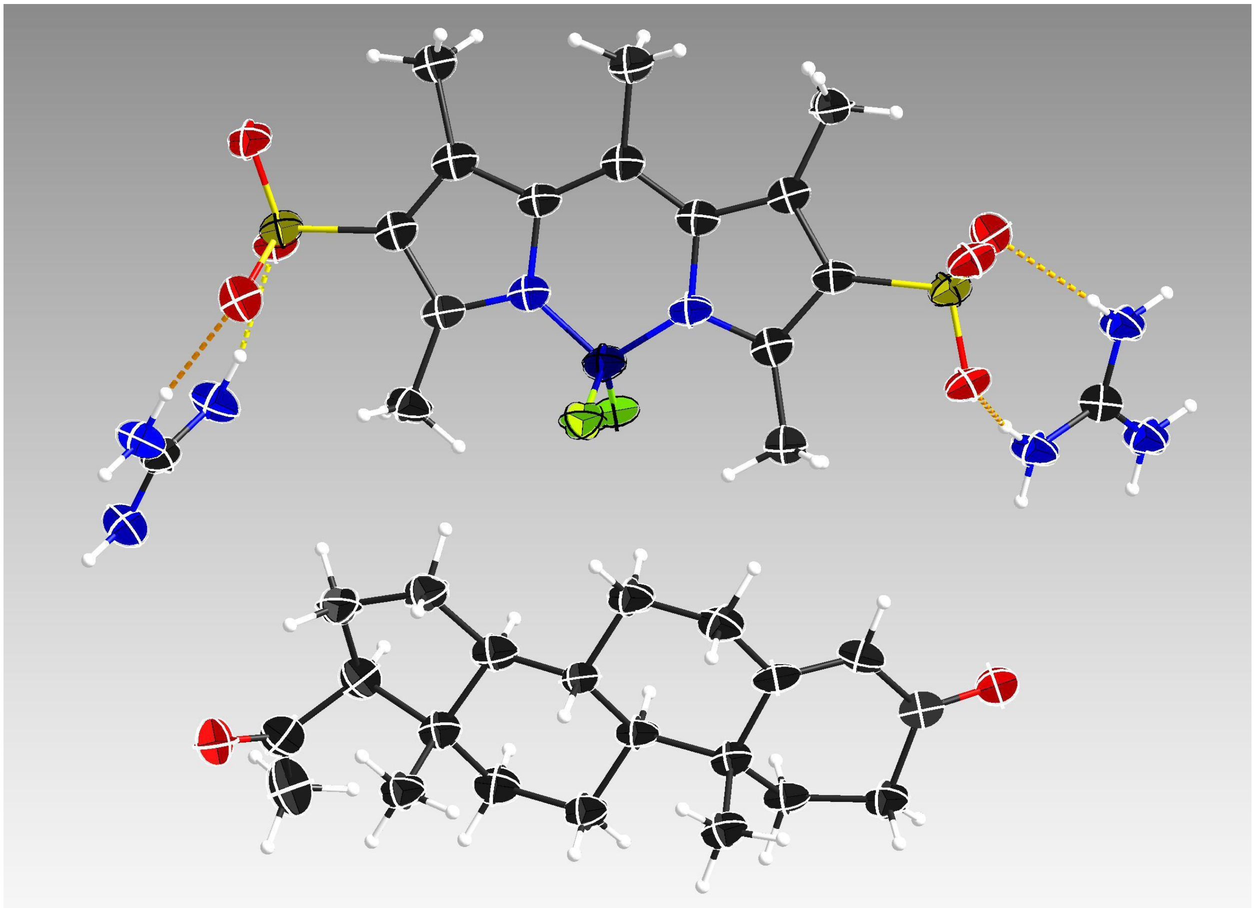

Supplementary Figure 11. Molecular structure of **7b** depicted as ellipsoids in 50% probability.

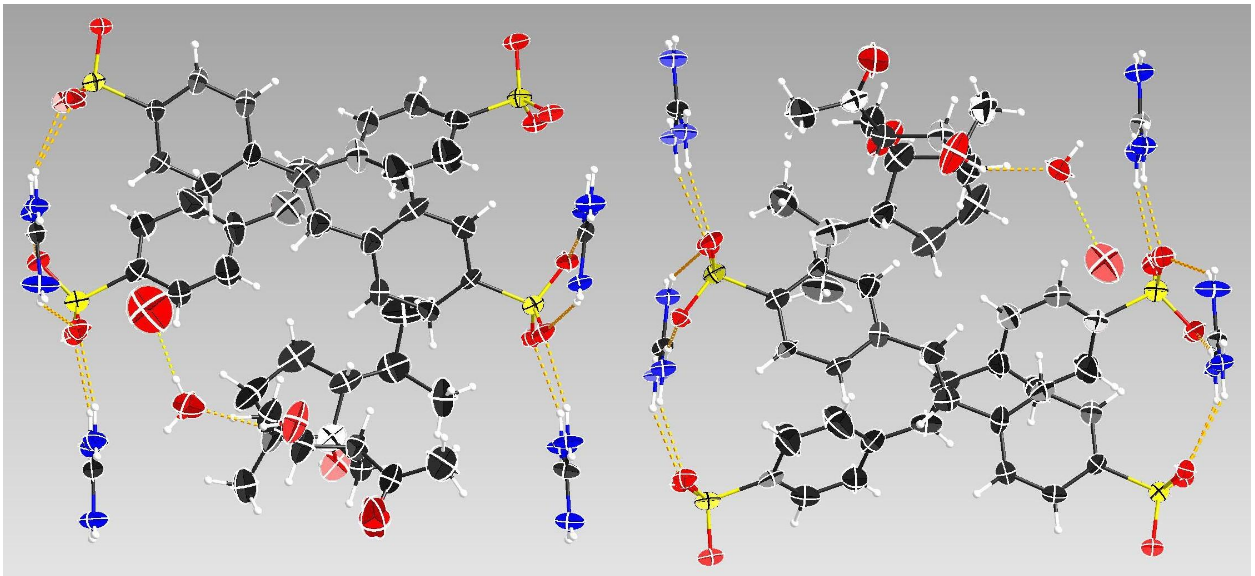

Supplementary Figure 12. Molecular structure of **8** depicted as ellipsoids in 50% probability.

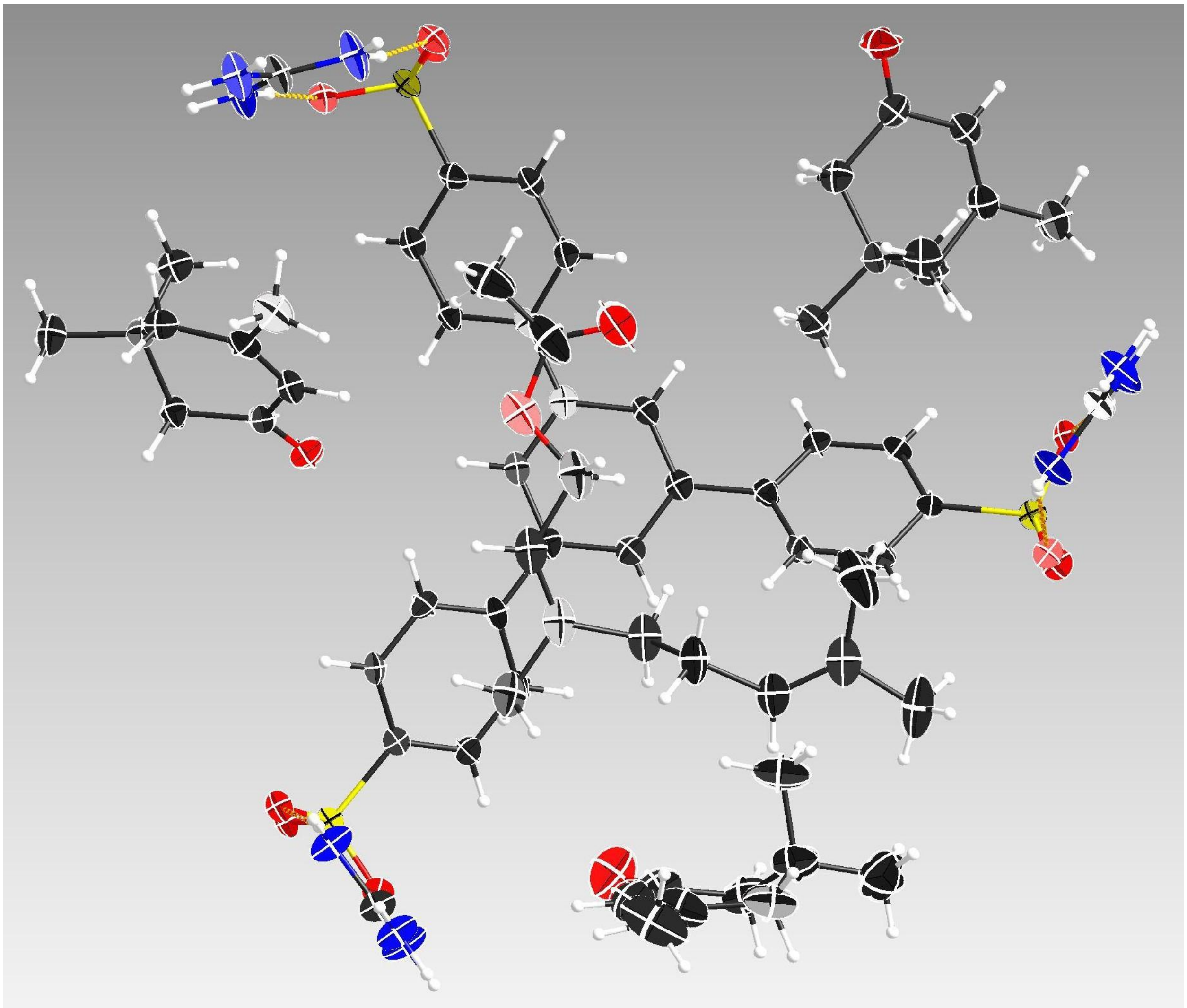

Supplementary Figure 13. Molecular structure of **9** depicted as ellipsoids in 50% probability.

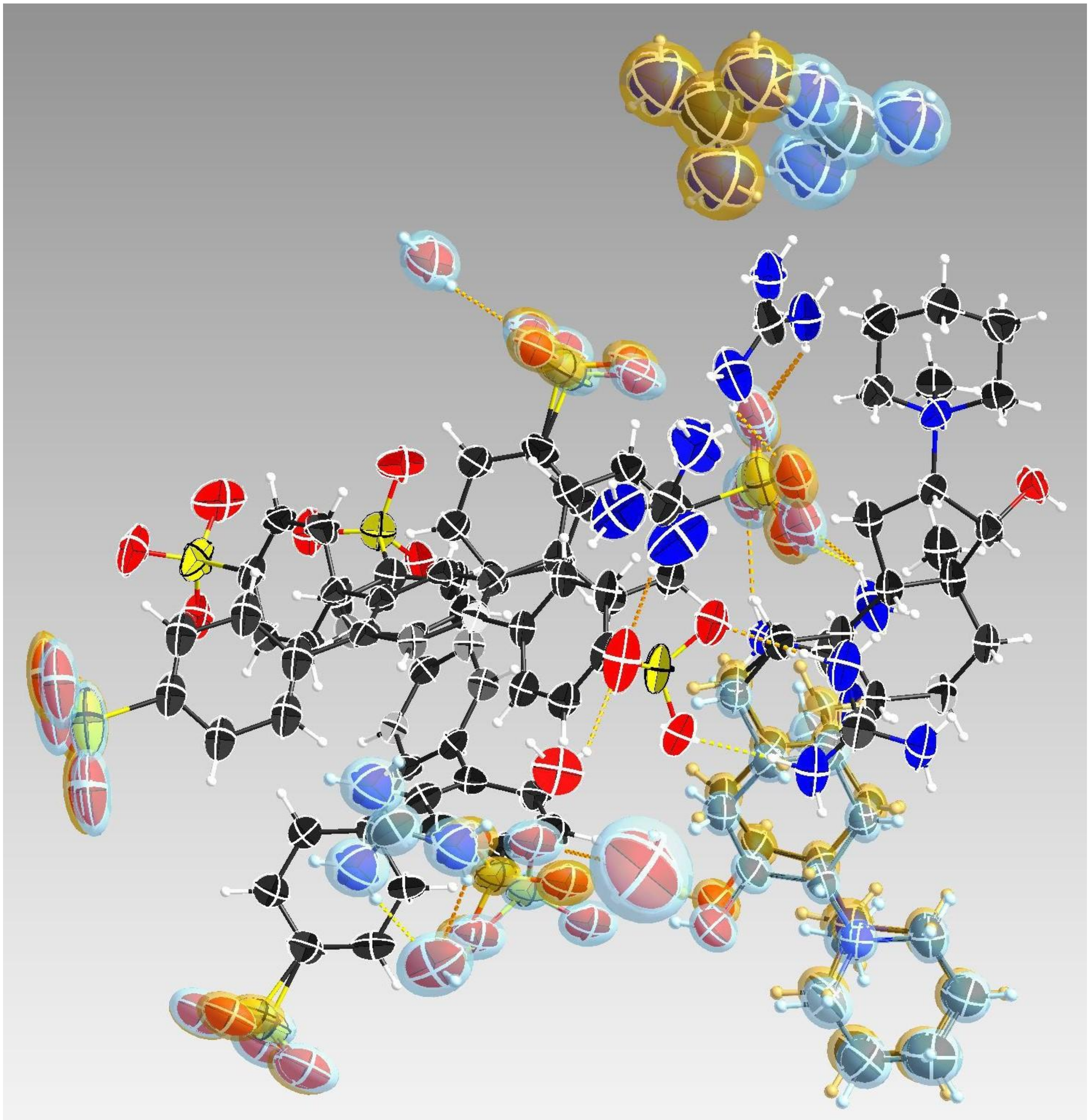

Supplementary Figure 14. Molecular structure of **10** depicted as ellipsoids in 50% probability.

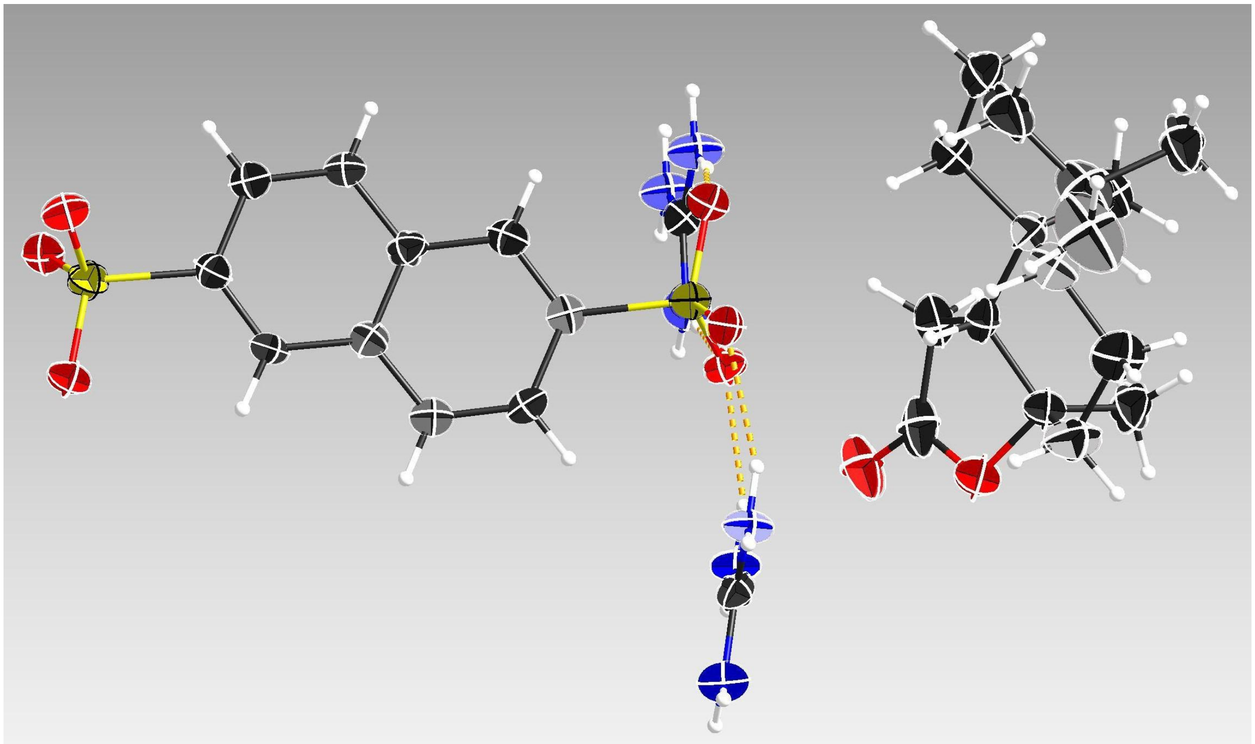

Supplementary Figure 15. Molecular structure of **5micro** depicted as ellipsoids in 50% probability.

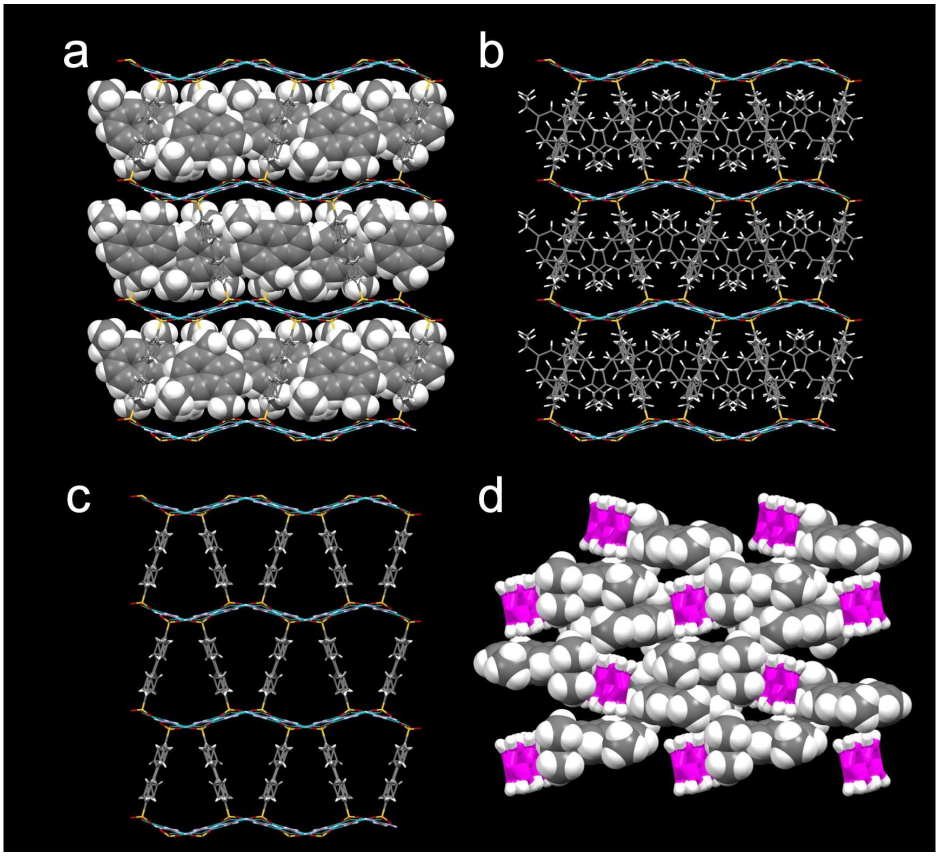

Supplementary Figure 16. Crystal structure of **1a** viewed down the *b*-axis. (a) Guest molecules are rendered as space-filled and the host framework as stick. (b) Guests and host framework both rendered as stick. (c) View of the host framework *ac* plane without guests, illustrating the zigzag brick framework. (d) View of the *ab* plane with guanidinium and sulfonate ions removed to reveal the packing of the organic residues (magenta) of the organosulfonate pillars and the guests.

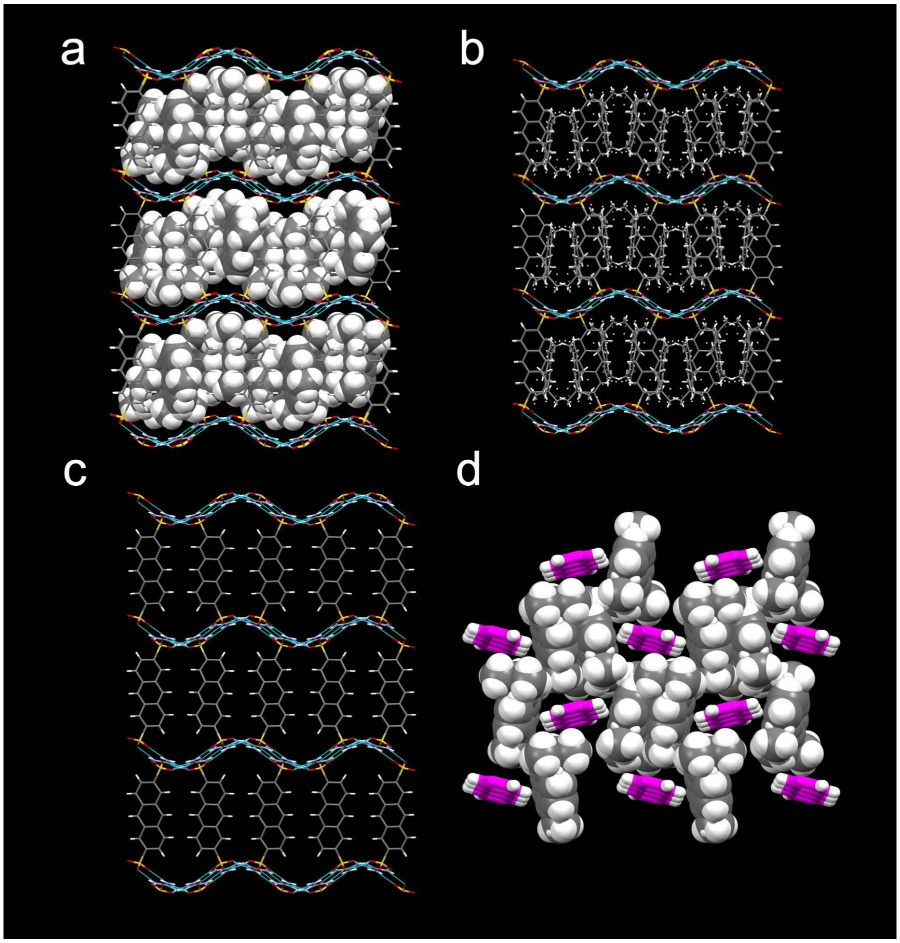

Supplementary Figure 17. Crystal structure of **1b** viewed down the *b*-axis. (a) Guest molecules are rendered as space-filled and the host framework as stick. (b) Guests and host framework both rendered as stick. (c) View of the host framework *ac* plane without guests, illustrating the zigzag brick framework. (d) View of the *ab* plane with guanidinium and sulfonate ions removed to reveal the packing of the organic residues (magenta) of the organosulfonate pillars and the guests.

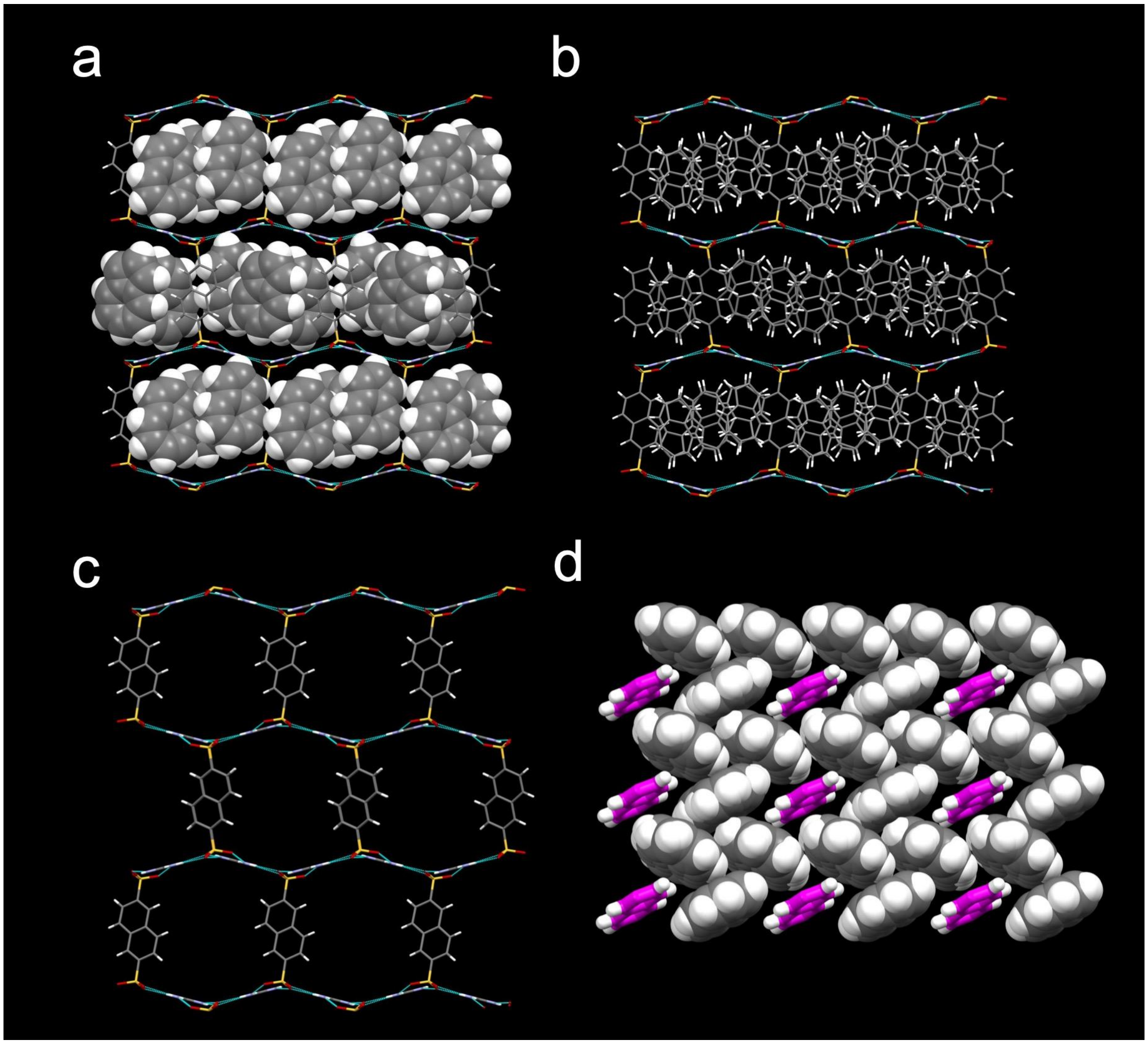

Supplementary Figure 18. Crystal structure of **2** viewed down the *a*-axis. (a) Guest molecules are rendered as space-filled and the host framework as stick. (b) Guests and host framework both rendered as stick. (c) View of the host framework *bc* plane without guests, illustrating the simple brick framework. (d) View of the *ac* plane with guanidinium and sulfonate ions removed to reveal the packing of the organic residues (magenta) of the organosulfonate pillars and the guests.

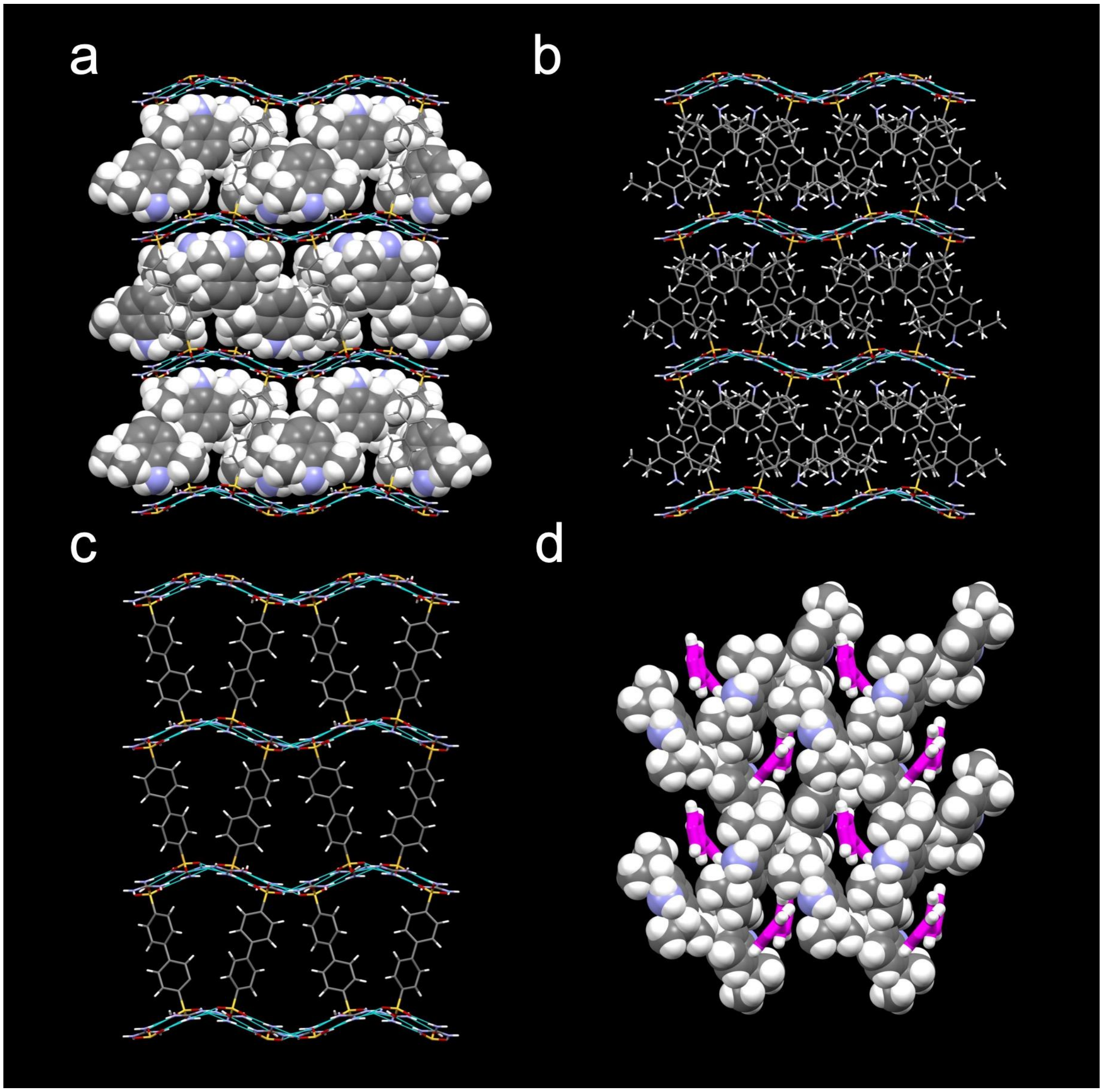

Supplementary Figure 19. Crystal structure of **3** viewed down the *a*-axis. (a) Guest molecules are rendered as space-filled and the host framework as stick. (b) Guests and host framework both rendered as stick. (c) View of the host framework *bc* plane without guests, illustrating the zigzag brick framework. (d) View of the *ab* plane with guanidinium and sulfonate ions removed to reveal the packing of the organic residues (magenta) of the organosulfonate pillars and the guests.

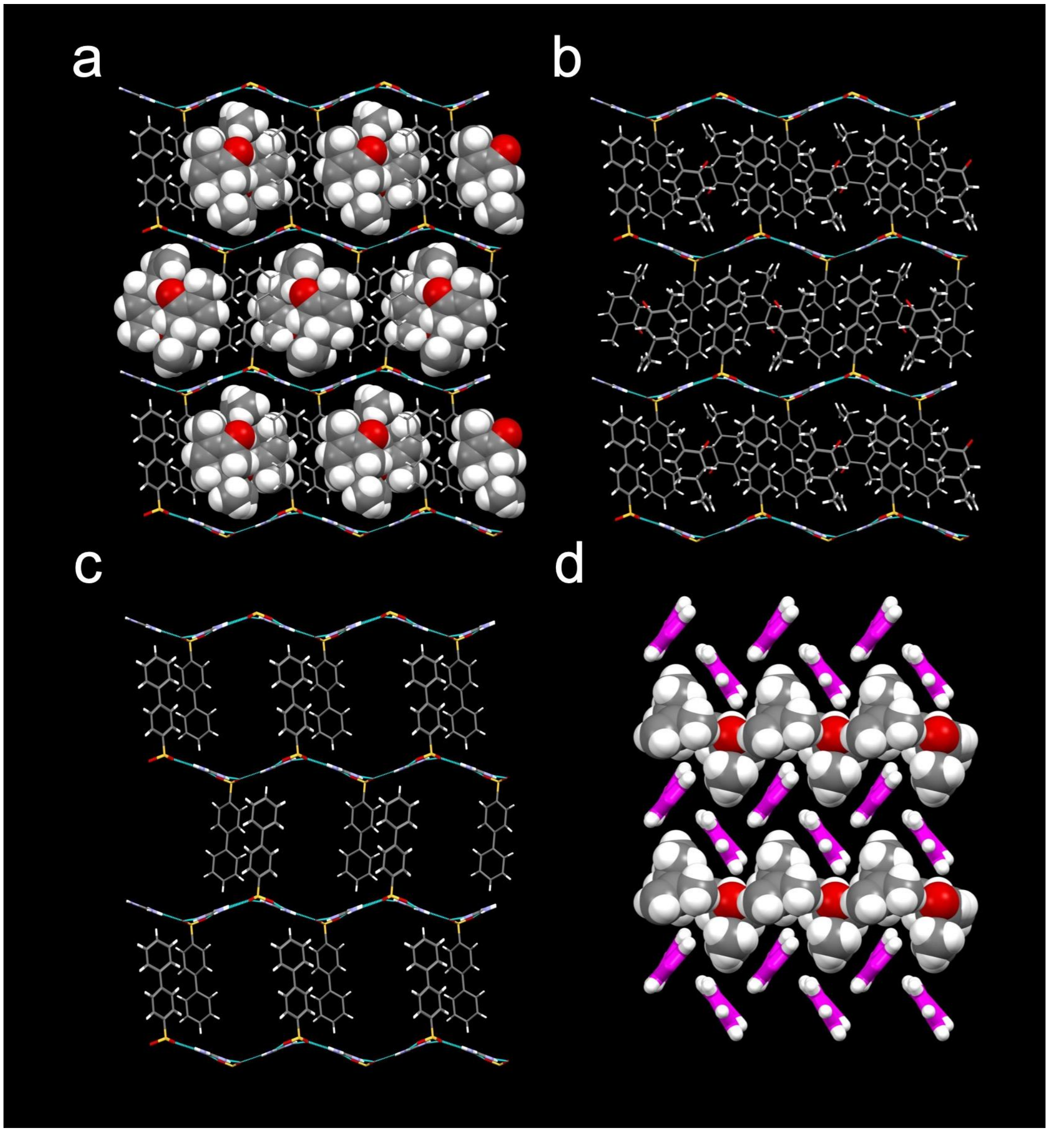

Supplementary Figure 20. Crystal structure of **4** viewed down the *a*-axis. (a) Guest molecules are rendered as space-filled and the host framework as stick. (b) Guests and host framework both rendered as stick. (c) View of the host framework *bc* plane without guests, illustrating the continuously layered framework. (d) View of the *ab* plane with guanidinium and sulfonate ions removed to reveal the packing of the organic residues (magenta) of the organosulfonate pillars and the guests.

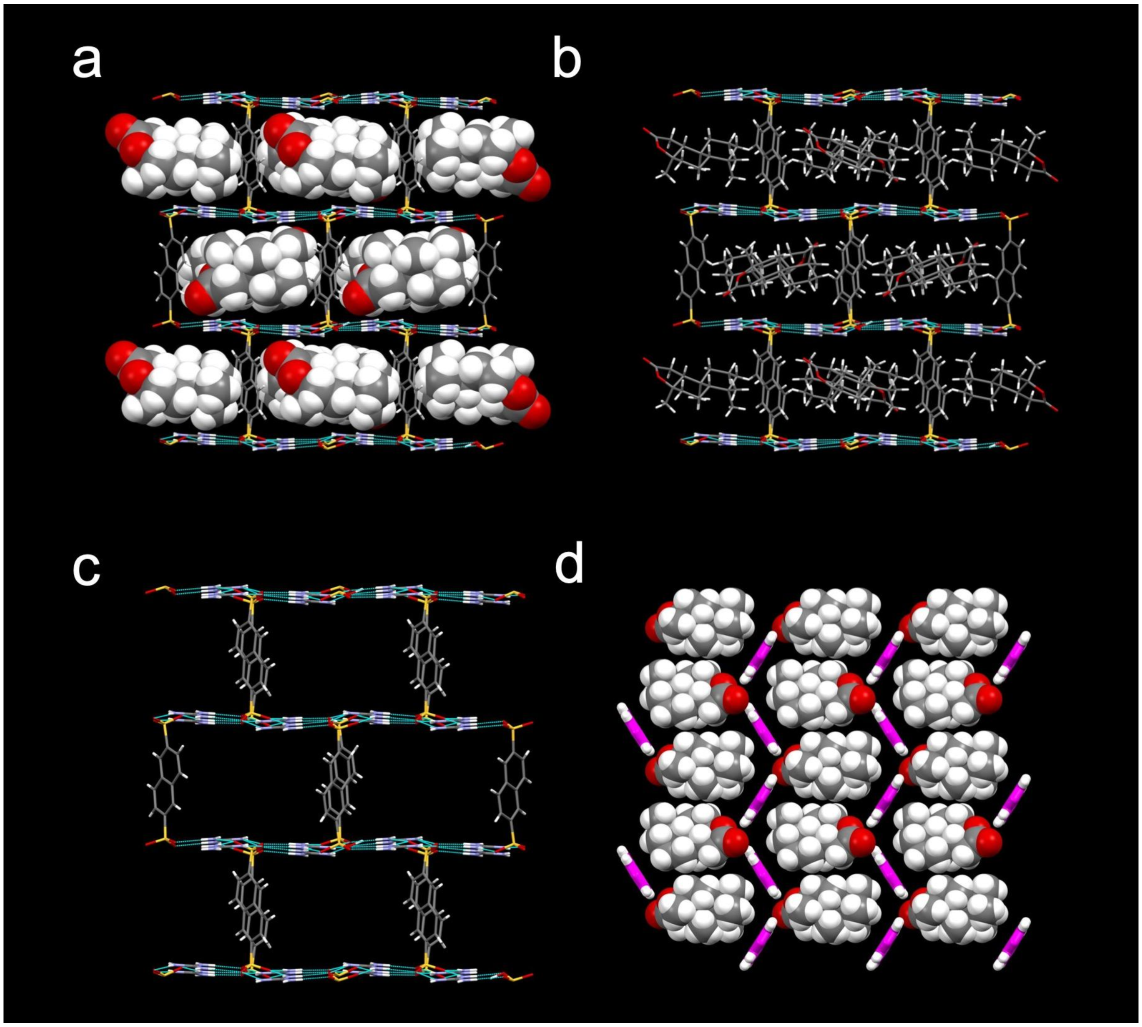

Supplementary Figure 21. Crystal structure of **5** viewed down the *b*-axis. (a) Guest molecules are rendered as space-filled and the host framework as stick. (b) Guests and host framework both rendered as stick. (c) View of the host framework *ac* plane without guests, illustrating the simple brick framework. (d) View of the *ab* plane with guanidinium and sulfonate ions removed to reveal the packing of the organic residues (magenta) of the organosulfonate pillars and the guests.

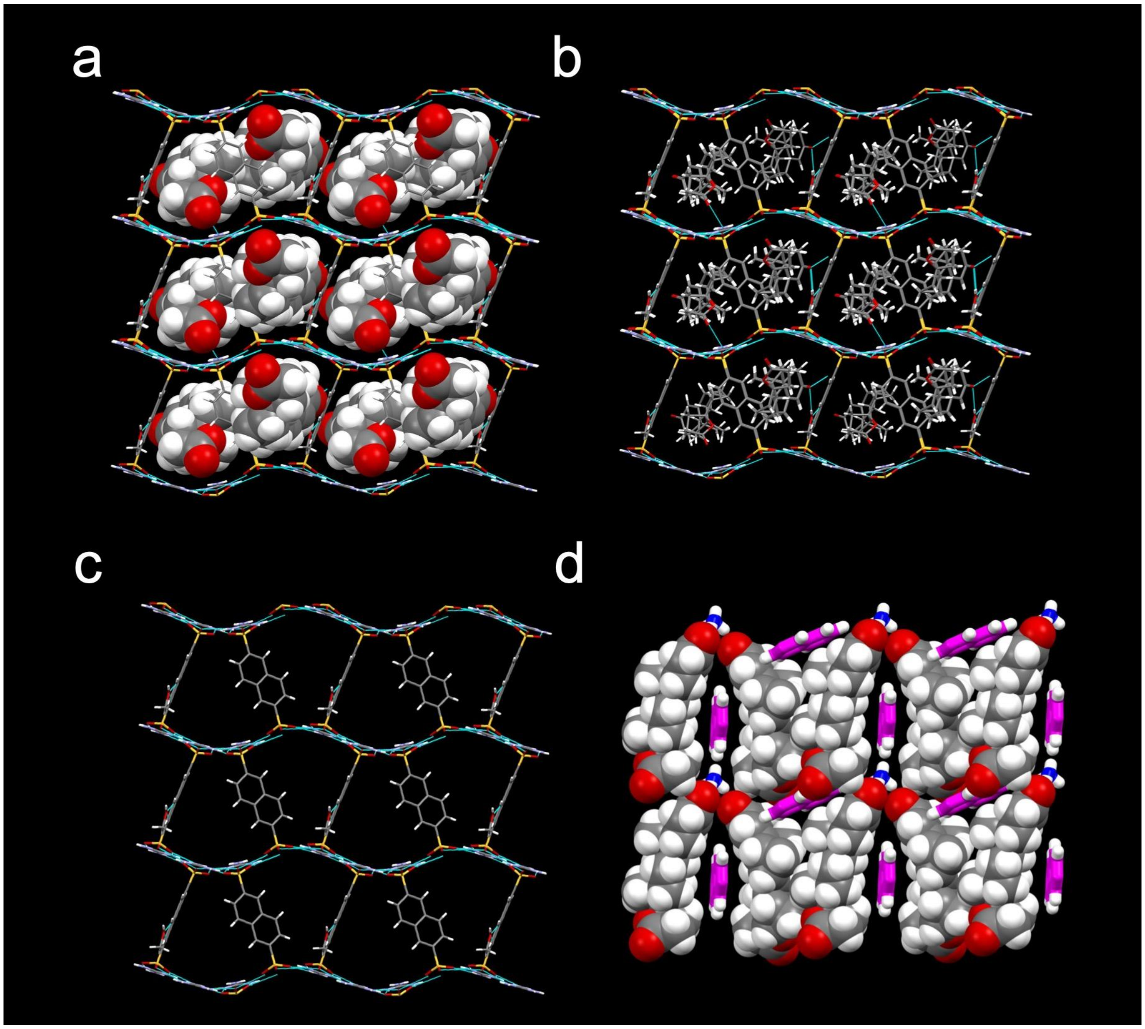

Supplementary Figure 22. Crystal structure of **6**. (a) Guest molecules are rendered as space-filled and the host framework as stick. (b) Guests and host framework both rendered as stick. (c) View of the host framework *ac* plane without guests, illustrating the zigzag brick framework. (d) View of the structure with guanidinium and sulfonate ions removed to reveal the packing of the organic residues (magenta) of the organosulfonate pillars and the guests. Solvent molecules are colored blue.

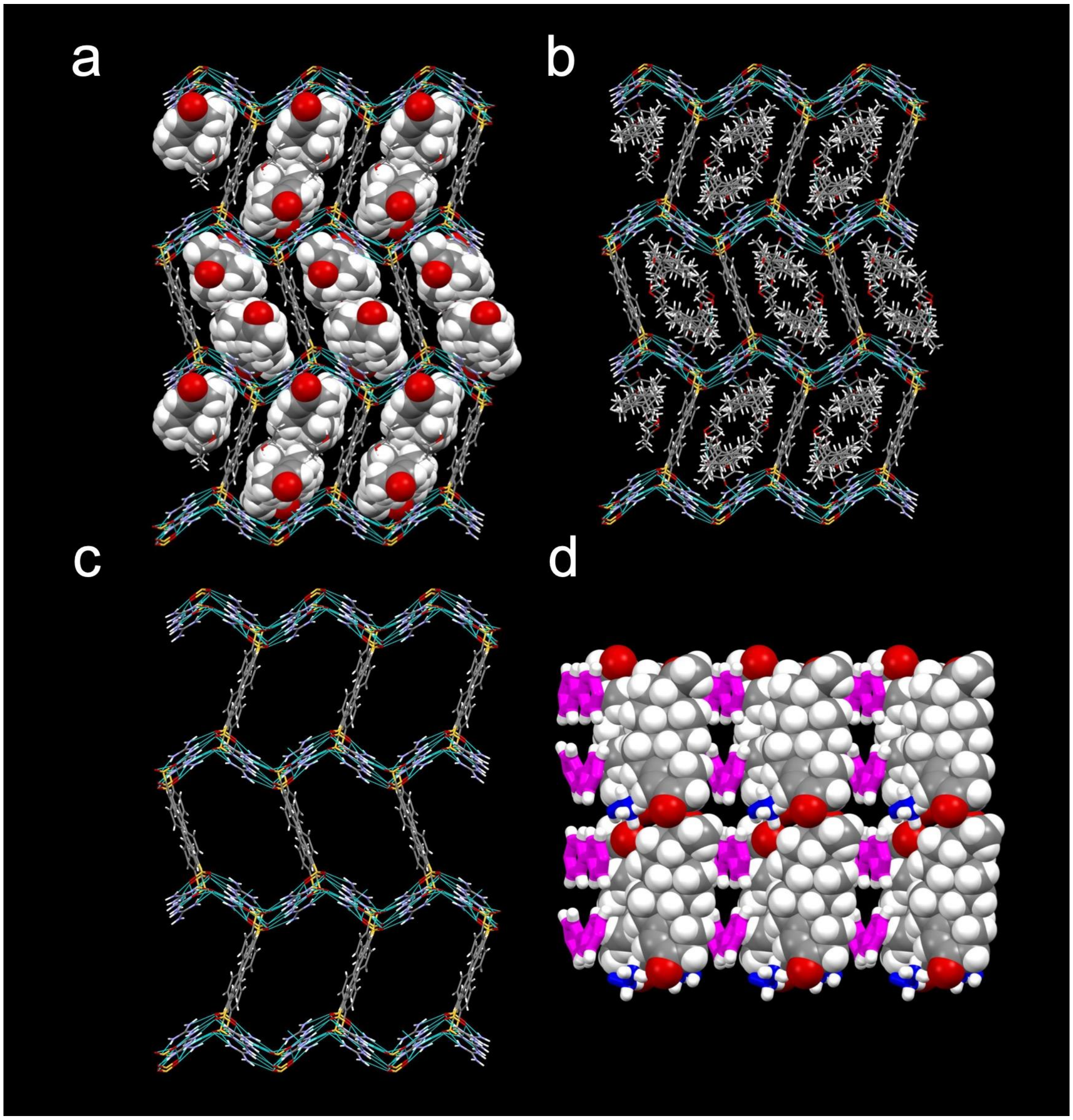

Supplementary Figure 23. Crystal structure of **7a** viewed down the *b*-axis. (a) Guest molecules are rendered as space-filled and the host framework as stick. (b) Guests and host framework both rendered as stick. (c) View of the host framework *ac* plane without guests, illustrating the simple brick framework. (d) View of the *ab* plane with guanidinium and sulfonate ions removed to reveal the packing of the organic residues (magenta) of the organosulfonate pillars and the guests. Solvent is colored blue.

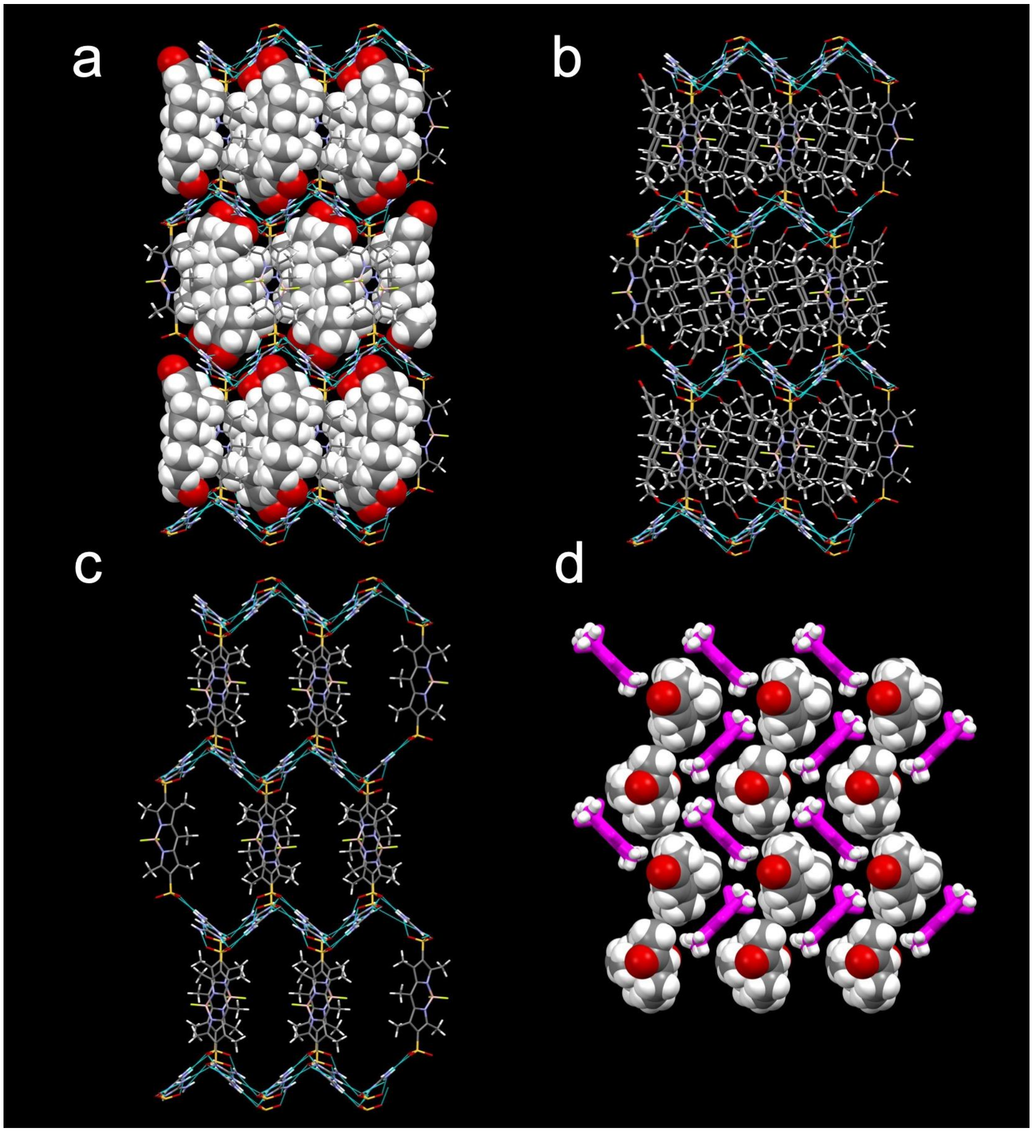

Supplementary Figure 24. Crystal structure of **7b** viewed down the *b*-axis. (a) Guest molecules are rendered as space-filled and the host framework as stick. (b) Guests and host framework both rendered as stick. (c) View of the host framework *ac* plane without guests, illustrating the simple brick framework. (d) View of the *ab* plane with guanidinium and sulfonate ions removed to reveal the packing of the organic residues (magenta) of the organosulfonate pillars and the guests.

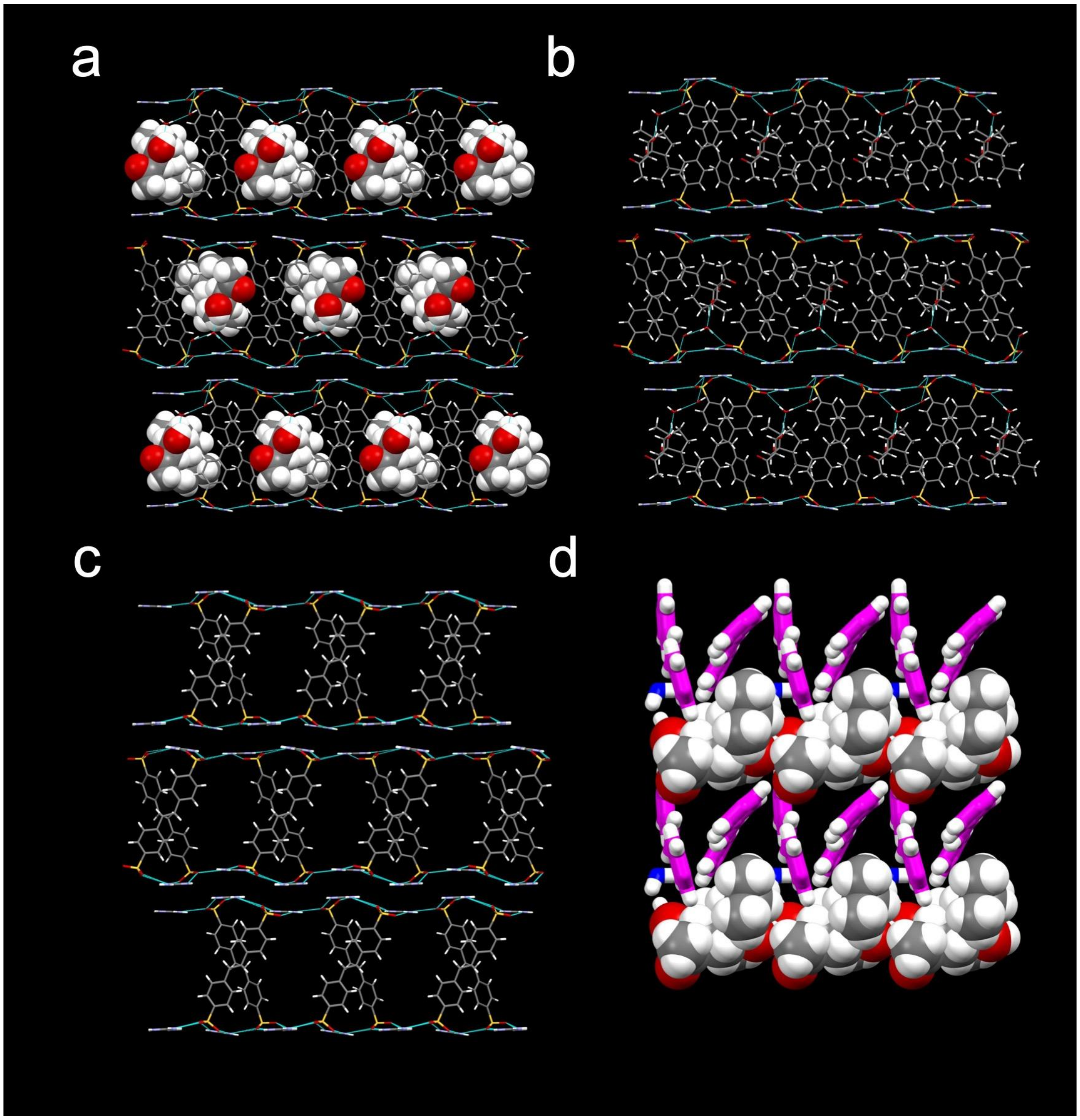

Supplementary Figure 25. Crystal structure of **8** viewed down the *a*-axis. (a) Guest molecules are rendered as space-filled and the host framework as stick. (b) Guests and host framework both rendered as stick. (c) View of the host framework *bc* plane without guests, illustrating the crisscross bilayer framework. (d) View of the *ab* plane with guanidinium and sulfonate ions removed to reveal the packing of the organic residues (magenta) of the organosulfonate pillars and the guests. Solvent molecules are colored blue.

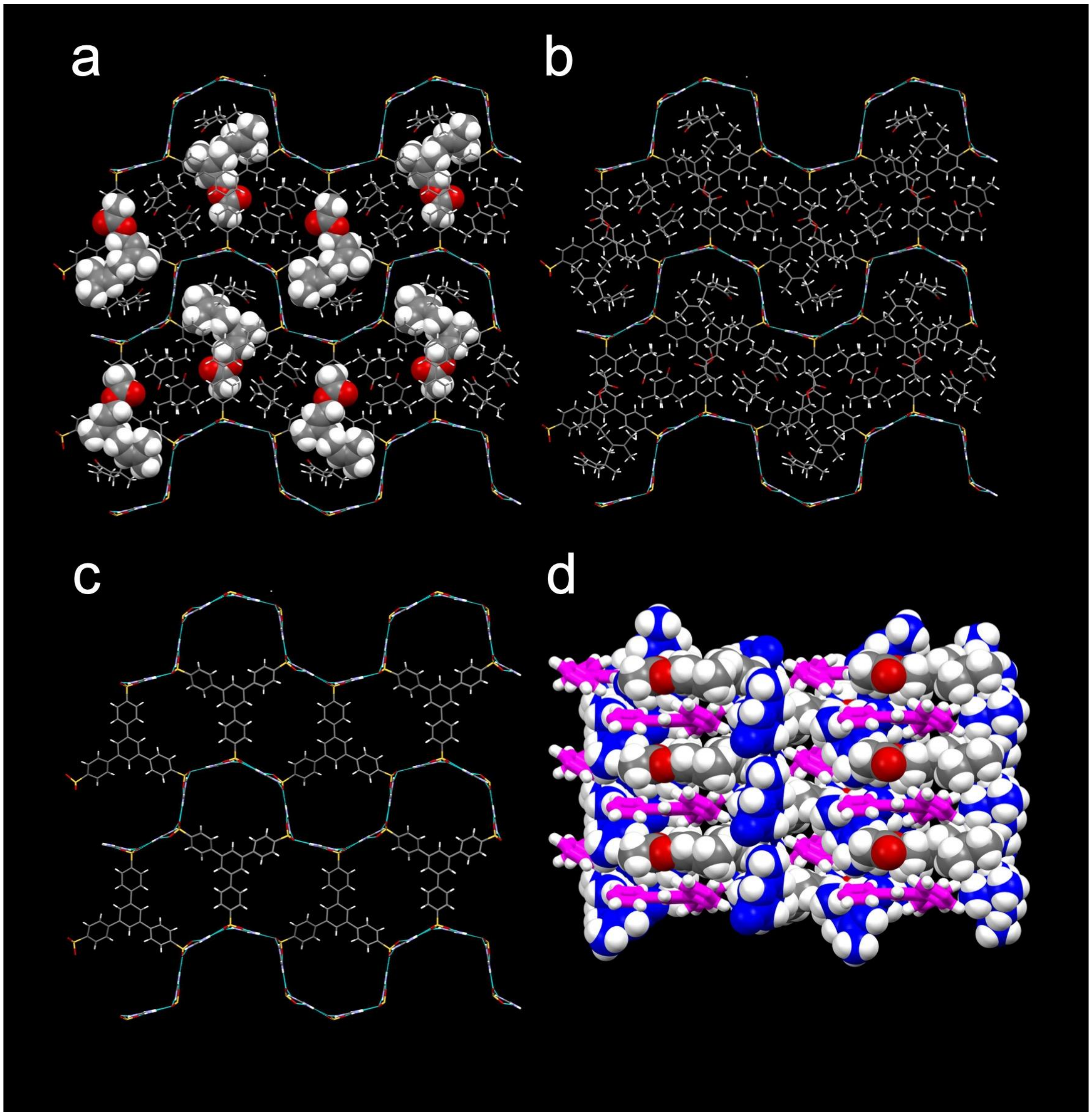

Supplementary Figure 26. Crystal structure of **9** viewed down the *a*-axis. (a) Guest molecules are rendered as space-filled and the host framework as stick. (b) Guests and host framework both rendered as stick. (c) View of the host framework *bc* plane without guests, illustrating the tricylindrical framework. (d) View of the *ab* plane with guanidinium and sulfonate ions removed to reveal the packing of the organic residues (magenta) of the organosulfonate pillars and the guests. Isophorone molecules are colored blue.

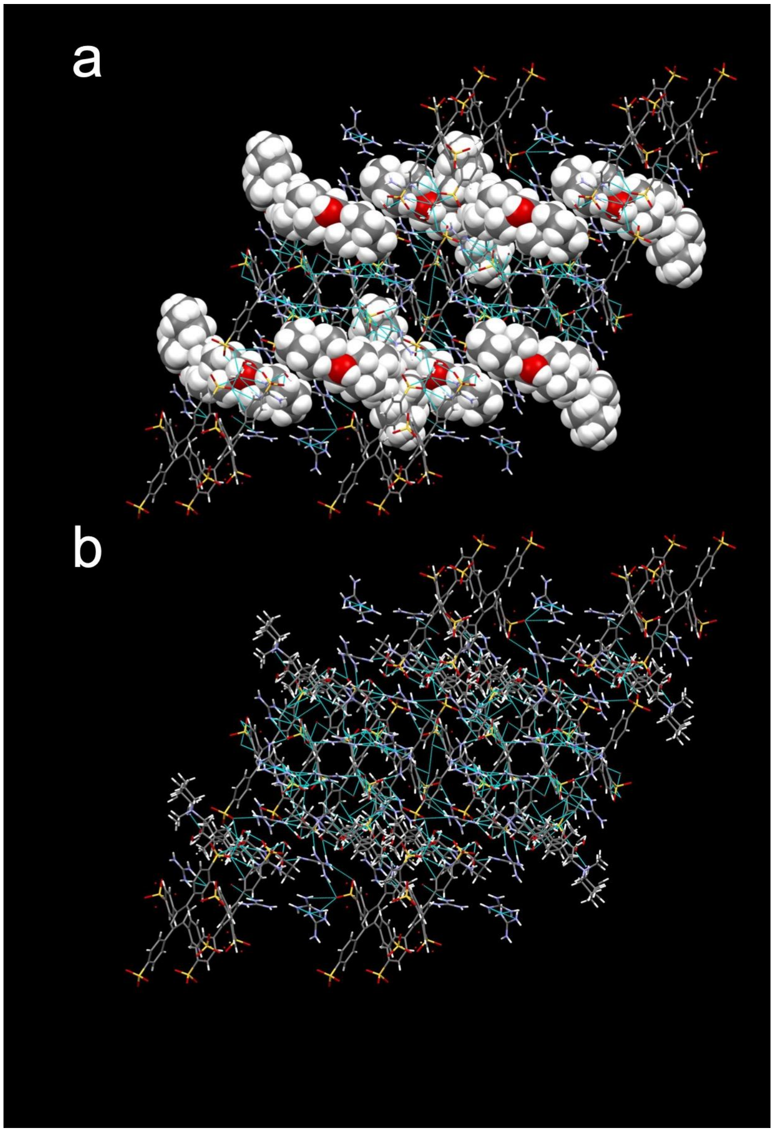

Supplementary Figure 27. Crystal structure of **10**. (a) Guest molecules are rendered as space-filled and the host framework as stick. (b) Guests and host framework both rendered as stick.

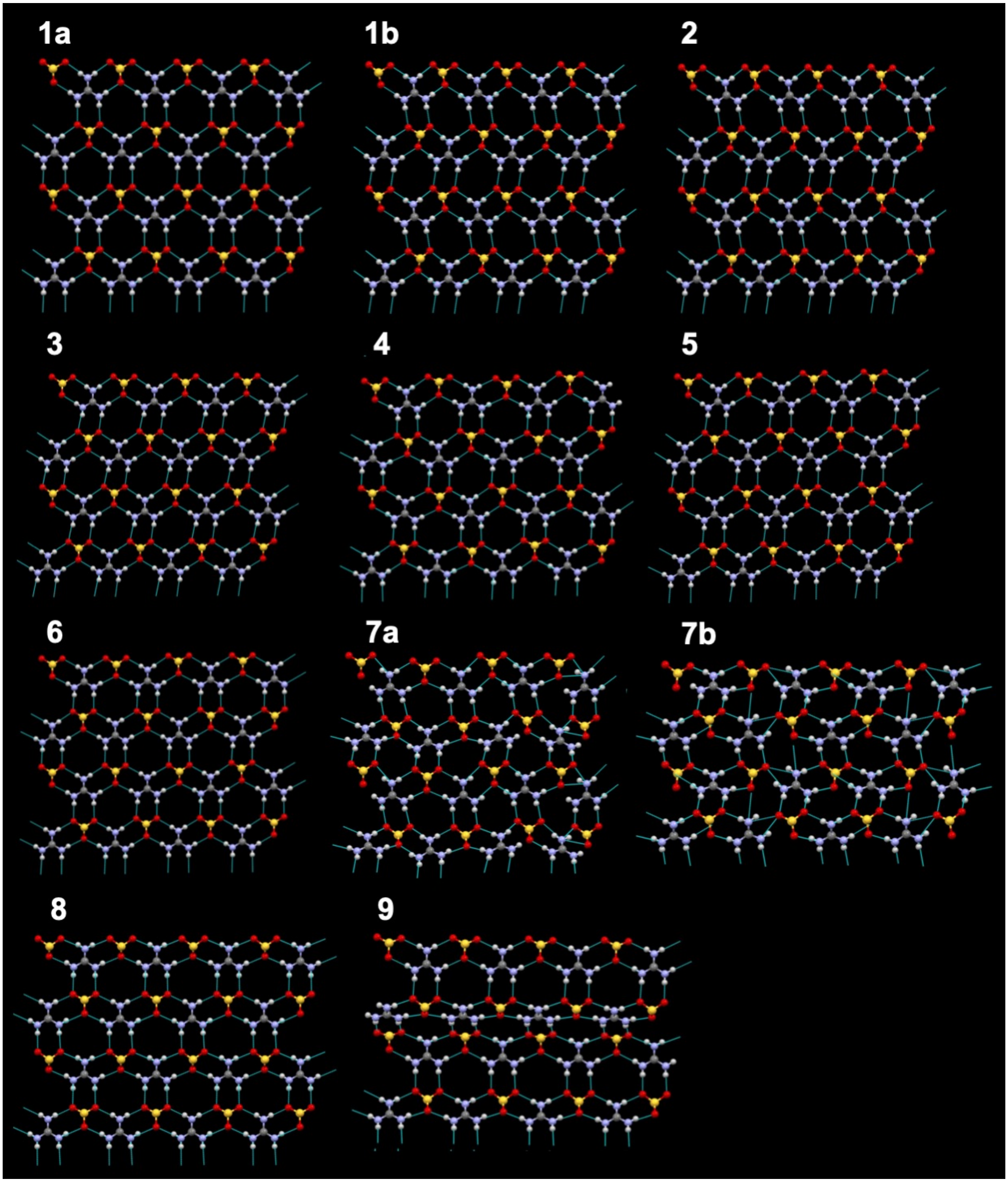

Supplementary Figure 28. Hydrogen bonded sheet motifs in compounds **1a** – **9**.

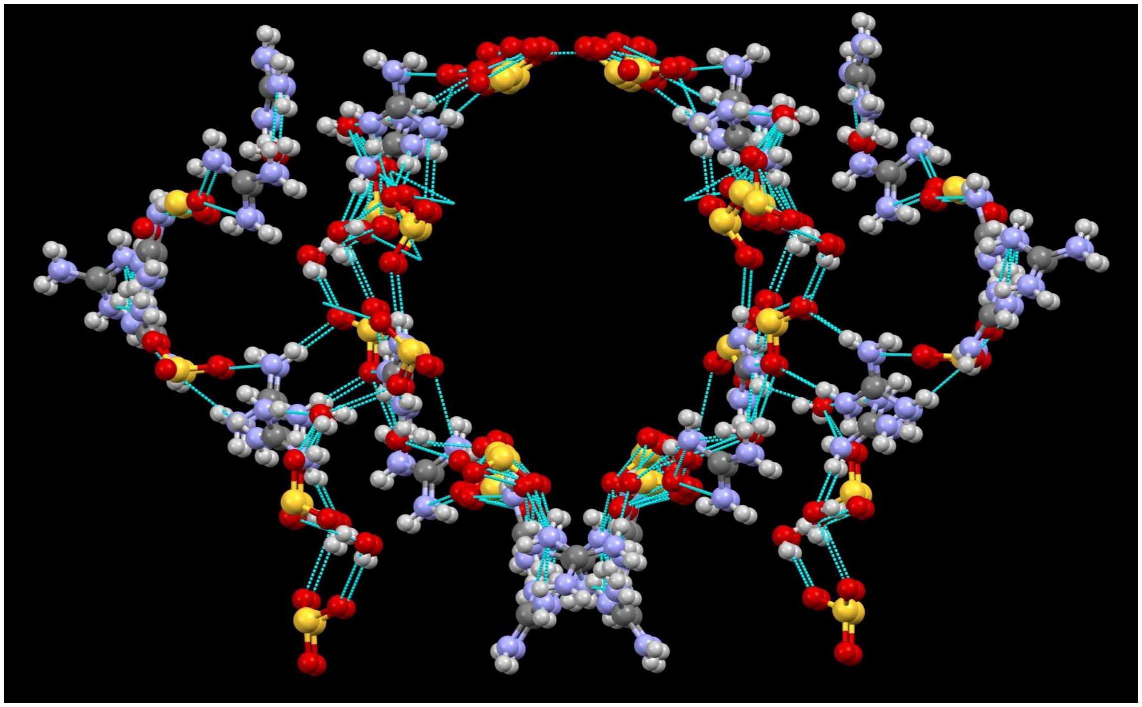

Supplementary Figure 29. Hydrogen bonds of guanidinium, water and sulfonate in compounds **10**.

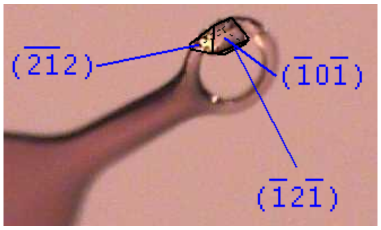

Supplementary Figure 30, A single crystal of (G_2NDS)⊃((3aR)-(+)-Sclareolide) grown from 5 micrograms of (3aR)-(+)-Sclareolide (**5micro**).

Supplementary References

1. Holman, K. T.; Martin, S. M.; Parker, D. P.; Ward, M. D. *J. Am. Chem. Soc.*, **2001**, *123*, 4421.
2. Soegiarto, A. C.; Comotti, A.; Ward, M. D. *J. Am. Chem. Soc.*, **2010**, *132*, 14603.
3. Suter, C. M.; Harrington, G. A. *J. Am. Chem. Soc.*, **1937**, *59*, 2575.
4. Schneider, H. J.; Schiestel, T.; Zimmermann, P. *J. Am. Chem. Soc.*, **1992**, *114*, 7698.
5. Mahmoudkhani, A. H.; Cote, A. P.; Shimizu, G. K. H. *Chem. Commun*. **2004**, 2678.
6. Horner, M. J.; Holman, K. T.; Ward, M. D. *J. Am. Chem. Soc.*, **2007**, *1299*, 14640.
7. Sheldrick, G. M. SHELXT. Program for solving small molecule structures, determining the space group and structure together, Universität Göttingen, Germany, **2014**.
8. Sheldrick, G. M. SHELXL. Program for refinement of crystal structures, Universität Göttingen, Germany, **2014**.
9. Hoshino, M.; Khutia, A.; Xing, H.; Inokuma, Y.; Fujita, M. *IUCrJ,* **2016**, *3*, 139.
10. Dittrich, B.; Fabbiani, F. P. A.; Henn, J.; Schmidt, M. U.; Macchi, P.; Meindl, K.; Spackman, M. A. *Acta Cryst.* **2018**, *B74*, 416.
11. Robertson, J. M.; Shearer, H. M. M.; Sim, G. A.; Watson, D. G. *Acta Cryst.* **1962**, *15*, 1.
12. Ramon, G.; Coleman, A. W.; Nassimbeni, L. R. *Cryst. Growth Des*. **2006**, *6*, 132.
13. Choi, D.; Lee, H.; Lee, J. J.; Jung, O.-J. *Cryst. Growth Des*. **2017**, *17*, 6677.
14. Gruber, T.; Fischer, C.; Seichter, W.; Bombicz, P.; Weber, E. *Cryst. Eng. Comm*. **2011**, *13*, 1422.
15. Zhou, W.; Hu, W.-X.; Xia, C.-N. *Acta Cryst*. **2006**, *E62*, o4132.
16. Parisi, M.; Freire, E.; Rusjan, M.; Moreno, J. M.; Bonadeo, H.; Vega, D. *J. Mol. Struct*. **2013**, 1040, 83.
17. Wang, W.; Sun, Q. M.; Li, S. S.; Guo, L. Q.; Li, H. *Powder Diffr.* **2016**, *31*, 63.
18. Wan, W.; Ma, G.; Wang, J.; Li, L.; Rao, S.; Zheng, C.; Jiang, H.; Deng, H.; Hao, *J. Org. Biomol. Chem.* **2013**, *11*, 6597.
19. Sagadevan, A.; Hwang, K. C.; Su, M.-D. *Nat. Comm.* **2017**, *8*, 1.
20. Campsteyn, H.; Dupont, L.; Dideberg, L. O. Structure cristalline et moléculaire de la progestérone $C_{21}H_{30}O_2$. *Acta Crystallogr., Sect. B*: *Struct. Crystallogr. Cryst. Chem.*, **1972**, *28*, 3032.
21. Serantoni, E. F.; Krajewski, A.; Mongiorgi, R.; Sanseverino, L. R.; Cameroni, R. *Cryst. Struct. Commun.*, **1975**, *4*, 189.
22. Friščić, T.; Lancaster, R. W.; Fábián, L.; Karamertzanis, P. G. *Proc. Natl. Acad. Sci.* **2010**, *107*, 13216.

23. Kula, J.; Bonikowski, R.; Staniszewska, M.; Krakowiak, A.; Wieczorek, M. W.; Majzner, W. R.L Bujacz, G. D. *Eur. J. Org. Chem.* **2002**, 1826.

24. Savage, D. S.; Cameron, A. F.; Ferguson, G.; Hannaway, C.; Mackay, I. R. *J. Chem. Soc. B*, **1971**, 410.
